# Supplementary material for: Analysis of CYP2C19 genetic variants with ischaemic events in UK patients prescribed clopidogrel in primary care: a retrospective cohort study
Source: BMJ Open. 2021 Dec 13;11(12):e053905. doi: 10.1136/bmjopen-2021-053905 (PMC8671970; doi:10.1136/bmjopen-2021-053905)
Supplement: Supplementary data [file bmjopen-2021-053905supp001.pdf]

SUPPLEMENTARY MATERIAL

Analysis of *CYP2C19* genetic variants with ischaemic events in UK patients prescribed clopidogrel in primary care: a retrospective cohort study

Pilling *et al*.

Contents

Supplementary Methods 1
Prescription data 1
Supplementary Table 1: prescription codes in UK Biobank 2
Supplementary Table 2: *CYP2C19* genotypes in UK Biobank 3
Supplementary Table 3: ICD-10 codes for Major Bleeding outcome 4
Supplementary Table 4: *CYP2C19* associations with incident outcomes stratified by *17 genotype 5

Supplementary Methods

Prescription data

To identify prescriptions of clopidogrel and aspirin we used the UK Biobank “Coding system lookups and mappings” file (https://biobank.ctsu.ox.ac.uk/crystal/refer.cgi?id=592). For clopidogrel we searched for “clopidogrel|plavix|grepid” to include other relevant drug names. For aspirin we searched for “aspirin|nu-seals|caprin|disprin” also including relevant alternate names. We then used the associated read 2 codes and drug names in the `gp_scripts` UK Biobank prescription data for the participants. We did not use BNF codes as the resolution was not great enough to uniquely identify individual drugs.

Pilling LC, *et al. BMJ Open* 2021; 11:e053905. doi: 10.1136/bmjopen-2021-053905

BMJ Publishing Group Limited (BMJ) disclaims all liability and responsibility arising from any reliance placed on this supplemental material which has been supplied by the author(s)

Supplementary Table 1: prescription codes in UK Biobank

medication	term_description	read_2	bnf_code
clopidogrel	clopidogrel	bu5..	02.09.00.00
	clopidogrel 300mg tablets	bu54.	02.09.00.00
	clopidogrel 75mg tablets	bu51.	02.09.00.00
	grepid 75mg tablets	bu55.	02.09.00.00
	plavix 300mg tablets	bu53.	02.09.00.00
	plavix 75mg tablets	bu52.	02.09.00.00
aspirin	*aspirin 100mg m/r tablets	bu29.	02.09.00.00
	*aspirin 300mg m/r tablets	bu2b.	02.09.00.00
	*aspirin 324mg e/c tablets	di1h.	04.07.01.00
	*aspirin 500mg m/r tablets	di19.	
	*aspirin 600mg e/c tablets	di1g.	04.07.01.00
	*aspirin 600mg tablets	di1i.	00.00.00.00
	*aspirin 75mg tablets	bu25.	02.09.00.00
	*caprin 300mg e/c tablets	di1k.	04.07.01.00
	*caprin 324mg e/c tablets	di1a.	04.07.01.00
	*caprin 75mg e/c tablets	bu2F.	02.09.00.00
	*disprin cv 100mg m/r tablets	bu28.	02.09.00.00
	*disprin cv 300mg m/r tablets	bu2a.	02.09.00.00
	aspirin 100mg effervescent tablets	bu21.	02.09.00.00
	aspirin 150mg suppositories	di1o.	04.07.01.00
	aspirin 162.5mg m/r capsules	bu2I.	02.09.00.00
	aspirin 300mg dispersible tablets	j112.	10.01.01.00
	aspirin 300mg e/c tablets	di1f.	04.07.01.00
	aspirin 300mg effervescent tablets	bu27.	02.09.00.00
	aspirin 300mg soluble tablets	di1m.	04.07.01.00
	aspirin 300mg suppositories	di1n.	04.07.01.00
	aspirin 300mg tablets	j111.	10.01.01.00
	aspirin 75mg dispersible tablets	di13.	
	aspirin 75mg dispersible tablets	bu23.	02.09.00.00
	aspirin 75mg e/c tablets	bu2B.	02.09.00.00
	aspirin 75mg soluble tablets	bu2c.	02.09.00.00
	aspirin [antiplatelet]	bu2..	02.09.00.00
	aspirin [central nervous system use]	di1..	04.07.01.00
	aspirin [cns] 300mg dispersible tablets	di12.	04.07.01.00
	aspirin [cns] 300mg tablets	di11.	04.07.01.00
	aspirin [musculoskeletal use]	j11..	10.01.01.00
	aspirin and the salicylates	j1...	10.01.01.00
	aspirin+metoclopramide 900mg/10mg/sachet powder	dl1b.	04.07.04.01
	aspirin+papaveretum 500mg/7.71mg dispersible tablets	diaG.	04.07.01.00
	aspirin/paracetamol/codeine tablets	dia1.	04.07.01.00
	dipyridamole+aspirin	bu4..	02.09.00.00
	dipyridamole+aspirin 200mg/25mg m/r capsules	bu41.	02.09.00.00
	disprin 300mg dispersible tablets	di1r.	04.07.01.00
	isosorbide mononitrate+aspirin	blm..	02.06.01.00
	isosorbide mononitrate+aspirin 60mg/150mg m/r tablets	blmy.	02.06.01.00
	isosorbide mononitrate+aspirin 60mg/75mg m/r tablets	blmz.	02.06.01.00
	nu-seals aspirin 300mg e/c tablets	di1c.	04.07.01.00
	nu-seals aspirin 600mg e/c tablets	di1d.	04.07.01.00
	nu-seals aspirin 75mg e/c tablets	bu2A.	02.09.00.00
	nu-seals cardio 75 e/c tablets	bu2G.	02.09.00.00

Supplementary Table 2: *CYP2C19* genotypes in UK Biobank

							UK Biobank					GnomAD	
CYP2C19	Function	RSID	CHR	BP	A1	A2	HWE_p	INFO	MAF1	MAF1 %	note	MAF2	MAF2 %
*2	LoF	rs4244285	10	96541616	G	A	0.9067	0.9998	0.1492	14.924	Imputed	0.1468	14.6800
*3	LoF	rs4986893	10	96540410	G	A	1.0000	1.0000	0.0001	0.006	Directly genotyped	0.0003	0.0264
*4	LoF	rs28399504	10	96522463	A	G	0.1615	0.7339	0.0022	0.216	Imputed	0.0025	0.2528
*5	LoF	rs56337013	10	96612495	C	T					Not in UKB imputed	0.0000	0.0008
*6	LoF	rs72552267	10	96535210	G	A					Not in UKB imputed	0.0003	0.0333
*7	LoF	rs72558186	10	96541756	T	C					Not in UKB imputed	0.0000	0.0000
*8	LoF	rs41291556	10	96535173	T	C	0.0415	1.0000	0.0030	0.304	Directly genotyped	0.0027	0.2710
*17	GoF	rs12248560	10	96521657	C	T	0.7279	0.9984	0.2154	21.539	Imputed	0.2314	23.1400

Function = LoF (Loss of Function, poor metaboliser), GoF (Gain of Function, rapid metaboliser)
BP = base position (hg19, build 37)
A1 = common allele
A2 = minor allele
HWE_p = Hardy-Weinberg deviation p-value
INFO = imputation quality score
MAF1 = minor allele frequency in UK Biobank Europeans
MAF2 = GnomAD European (non-Finnish) population minor allele frequency. GnomAD data can be viewed using URLs https://gnomad.broadinstitute.org/variant/rs4244285 and substituting the RSID for the appropriate variant.

Supplementary Table 3: ICD-10 codes for Major Bleeding outcome

From DOI 10.1111/jep.13400

Code	Text Descriptor	Event
D62	Acute post-haemorrhagic anaemia	Other major bleeding
H35.6	Retinal haemorrhage	Other major bleeding
H43.1	Vitreous haemorrhage	Other major bleeding
I60.*	Subarachnoid haemorrhage	Intracranial bleeding
I61.*	Intracerebral haemorrhage	Intracranial bleeding
I62.*	Other non-traumatic intracranial haemorrhage	Intracranial bleeding
I85.0	Oesophageal varices with bleeding	GI bleeding
J94.2	Haemothorax	Other major bleeding
K25.0	Gastric ulcer, acute with haemorrhage	GI bleeding
K25.2	Gastric ulcer, acute with both haemorrhage and perforation	GI bleeding
K25.4	Gastric ulcer, chronic or unspecified with haemorrhage	GI bleeding
K25.6	Chronic or unspecified with both haemorrhage and perforation	GI bleeding
K26.0	Duodenal ulcer, acute with haemorrhage	GI bleeding
K26.2	Duodenal ulcer, acute with both haemorrhage and perforation	GI bleeding
K26.4	Duodenal ulcer, chronic or unspecified with haemorrhage	GI bleeding
K26.6	Chronic or unspecified with both haemorrhage and perforation	GI bleeding
K27.0	Peptic ulcer, acute with haemorrhage	GI bleeding
K27.2	Peptic ulcer, acute with both haemorrhage and perforation	GI bleeding
K27.4	Peptic ulcer, chronic or unspecified with haemorrhage	GI bleeding
K27.6	Chronic or unspecified with both haemorrhage and perforation	GI bleeding
K28.0	Gastrojejunal ulcer, acute with haemorrhage	GI bleeding
K28.2	Acute with both haemorrhage and perforation	GI bleeding
K28.6	Chronic or unspecified with both haemorrhage and perforation	GI bleeding
K29.0	Acute haemorrhagic gastritis	GI bleeding
K66.1	Haemoperitoneum	GI bleeding
K92.0	Haematemesis	GI bleeding
K92.1	Melaena	GI bleeding
N02.*	Recurrent and persistent haematuria	Other major bleeding
N92.4	Excessive bleeding in the premenopausal period	Other major bleeding
R04.*	Haemorrhage from respiratory passages	Other major bleeding
R58	Haemorrhage, not elsewhere classified	Other major bleeding

Note: we excluded N95.0, K62.5 and R31.* from our criteria as these were common (>10,000 participants ever diagnosed in whole dataset) and therefore deemed not specific to Major Bleeding for the purpose of this analysis.

Supplementary Table 4: *CYP2C19* associations with incident outcomes stratified by *17 genotype

Outcome / CYP2C19 genotype	N	N cases	Person-years	HR	95% CIs		*p*
Ischemic stroke							
Normal (*1/*1)	2,948	37	7,208				
Intermediate/poor (any *2-*8)	1,668	33	3,919	1.61	1.01	2.58	0.047
Poor or rapid heterozygote (*2–*8/*17)	476	8	1,191	1.25	0.58	2.69	0.570
Rapid (any *17)	2,385	32	6,039	0.99	0.62	1.59	0.970
Total	*7,477*	*110*	*18,358*				
Myocardial infarction							
Normal (*1/*1)	2,905	707	5,403				
Intermediate/poor (any *2-*8)	1,634	442	2,805	1.16	1.03	1.30	0.017
Poor or rapid heterozygote (*2–*8/*17)	466	112	904	0.98	0.81	1.20	0.880
Rapid (any *17)	2,354	562	4,691	0.95	0.85	1.06	0.330
Total	*7,359*	*1823*	*13,803*				

Analysis of European-ancestry participants with >1 clopidogrel prescription in the available GP prescribing data. Participants excluded if clopidogrel prescribing frequency was less than once every 2 months. Events <1 week after first clopidogrel prescription are excluded. Events occurring after the last known date of clopidogrel prescription are also excluded. HR=Hazard Ratios from Cox's proportional hazards regression models adjusted for age at first clopidogrel prescription, sex, and genetic principal components of ancestry 1-10. CI = Confidence Intervals.

Pilling LC, *et al. BMJ Open* 2021; 11:e053905. doi: 10.1136/bmjopen-2021-053905
