# Supplementary material for: Analysis of CYP2C19 genetic variants with ischaemic events in UK patients prescribed clopidogrel in primary care: a retrospective cohort study
Source: BMJ Open. 2021 Dec 13;11(12):e053905. doi: 10.1136/bmjopen-2021-053905 (PMC8671970; doi:10.1136/bmjopen-2021-053905)
Supplement: Author's manuscript [file bmjopen-2021-053905.draft_revisions.pdf]

BMJ Open is committed to open peer review. As part of this commitment we make the peer review history of every article we publish publicly available.

When an article is published we post the peer reviewers' comments and the authors' responses online. We also post the versions of the paper that were used during peer review. These are the versions that the peer review comments apply to.

The versions of the paper that follow are the versions that were submitted during the peer review process. They are not the versions of record or the final published versions. They should not be cited or distributed as the published version of this manuscript.

BMJ Open is an open access journal and the full, final, typeset and author-corrected version of record of the manuscript is available on our site with no access controls, subscription charges or pay-per-view fees (http://bmjopen.bmj.com).

If you have any questions on BMJ Open's open peer review process please email info.bmjopen@bmj.com

BMJ Open

Genetic variation in activating clopidogrel: long-term outcomes in a large community cohort

Journal:	*BMJ Open*
Manuscript ID	bmjopen-2021-053905
Article Type:	Original research
Date Submitted by the Author:	01-Jun-2021
Complete List of Authors:	Pilling, Luke; University of Exeter, Epidemiology and Public Health<br>Türkmen, Deniz; University of Exeter, Epidemiology and Public Health<br>Fullalove, Hannah; University of Exeter, Epidemiology and Public Health<br>Atkins, Janice; University of Exeter,<br>Delgado, Joao; University of Exeter, College of Medicine and Health<br>Kuo, Chia-Ling; UConn Health<br>Kuchel, George; University of Connecticut,<br>Ferrucci, Luigi; Harbor Hospital, Baltimore, National Institute on Aging NIA-ASTRA Unit<br>Bowden, Jack; University of Exeter, Epidemiology and Public Health<br>Masoli, Jane; University of Exeter, Epidemiology and Public Health<br>Melzer, David; University of Exeter, Epidemiology and Public Health
Keywords:	Myocardial infarction < CARDIOLOGY, EPIDEMIOLOGY, GENETICS, Stroke < NEUROLOGY, PREVENTIVE MEDICINE

SCHOLARONE™
Manuscripts

I, the Submitting Author has the right to grant and does grant on behalf of all authors of the Work (as defined in the below author licence), an exclusive licence and/or a non-exclusive licence for contributions from authors who are: i) UK Crown employees; ii) where BMJ has agreed a CC-BY licence shall apply, and/or iii) in accordance with the terms applicable for US Federal Government officers or employees acting as part of their official duties; on a worldwide, perpetual, irrevocable, royalty-free basis to BMJ Publishing Group Ltd ("BMJ") its licensees and where the relevant Journal is co-owned by BMJ to the co-owners of the Journal, to publish the Work in this journal and any other BMJ products and to exploit all rights, as set out in our licence.

The Submitting Author accepts and understands that any supply made under these terms is made by BMJ to the Submitting Author unless you are acting as an employee on behalf of your employer or a postgraduate student of an affiliated institution which is paying any applicable article publishing charge ("APC") for Open Access articles. Where the Submitting Author wishes to make the Work available on an Open Access basis (and intends to pay the relevant APC), the terms of reuse of such Open Access shall be governed by a Creative Commons licence – details of these licences and which Creative Commons licence will apply to this Work are set out in our licence referred to above.

Other than as permitted in any relevant BMJ Author's Self Archiving Policies, I confirm this Work has not been accepted for publication elsewhere, is not being considered for publication elsewhere and does not duplicate material already published. I confirm all authors consent to publication of this Work and authorise the granting of this licence.

For peer review only

Genetic variation in activating clopidogrel: long-term outcomes in a large community cohort

Luke C. Pilling[1,2], Deniz Türkmen[1], Hannah Fullalove[1], Janice L. Atkins[1], João Delgado[1], Chia-Ling Kuo[3], George A. Kuchel[2], Luigi Ferrucci[4], Jack Bowden[5], Jane A.H. Masoli[1,6], David Melzer[1,2]

1. Epidemiology and Public Health Group, College of Medicine and Health, University of Exeter, Exeter, UK

2. UConn Center on Aging, University of Connecticut, Farmington, CT, USA

3. Connecticut Convergence Institute for Translation in Regenerative Engineering, University of Connecticut, CT, USA

4. National Institute on Aging, Baltimore, MD, USA

5. Exeter Diabetes Group (ExCEED), College of Medicine and Health, University of Exeter, Exeter, UK

6. Department of Healthcare for Older People, Royal Devon and Exeter Hospital, Barrack Road, Exeter, UK.

Corresponding author

Dr Luke Pilling (L.Pilling@exeter.ac.uk)

Epidemiology and Public Health Group, University of Exeter, College House, St. Luke's Campus, Exeter, EX1 2LU, UK.

Word count: (Introduction/Methods/Results/Discussion) 4,151

For peer review only

Abstract

Objective To determine whether *CYP2C19* Loss of Function (LoF) alleles (which reduce clopidogrel pro-drug activation to the active form in the liver) increase risk of ischaemic stroke and myocardial infarction (MI) in UK primary care patients prescribed clopidogrel.

Design Retrospective cohort analysis.

Setting Primary care practices in the UK from January 1999 to September 2017.

Participants 7,483 European-ancestry adults from the UK Biobank study with genetic and linked primary care data, aged 36 to 79 years at time of first clopidogrel prescription.

Interventions Clopidogrel prescription in primary care, mean duration 2.6 years (range 2 months to 18 years)

Main outcome measure Hospital inpatient-diagnosed ischaemic stroke, MI or angina whilst treated with clopidogrel.

Results 28.7% of participants carried at least one C*YP2C19* LoF variant. LoF carriers had higher rates of incident ischaemic stroke whilst treated with clopidogrel compared to those without the variants (8 per 1,000 person-years versus 5.2 per 1,000 person-years; Hazard Ratio 1.53: 95% Confidence Intervals 1.04 to 2.26, p=0.031). In lifetables using observed incidence rates, 22.5% (95%CI 14.4% to 34.0%) of *CYP2C19* LoF carriers on clopidogrel were projected to develop an ischaemic stroke by age 79 (oldest age in the study), compared to 15.4% (95%CI 11.4% to 20.5%) in non-carriers, i.e. 7.1% excess stroke incidence in LoF carriers by age 79. Adjustment for aspirin co-prescription produced similar estimates. LoF carriers also had increased incident MI risk (HR 1.14: 95%CI 1.04 to 1.26, p=0.008).

Conclusions A substantial proportion of the UK population carry genetic variants that reduce metabolism of clopidogrel to its active form. In family practice patients on clopidogrel, *CYP2C19* LoF variants are associated with substantially higher incidence of ischaemic events. Genotype-guided selection of antiplatelet medications may improve outcomes in patients carrying *CYP2C19* genetic variants.

For peer review only - http://bmjopen.bmj.com/site/about/guidelines.xhtml

Article Summary

Strengths and limitations of this study

Genetic variants are known to modify the effectiveness of clopidogrel antiplatelet treatment in the acute setting, but the magnitude of impact in family practice is unclear, especially during long treatments.

We compared incidence of ischaemic events between carriers of *CYP2C19* loss of function variants in a large UK cohort prescribed with clopidogrel or up to 18 years.

Though the UK Biobank cohort is very large, the mean age at first clopidogrel prescription was 63 so the absolute incidence rates of ischaemic events in the whole cohort were low.

For peer review only

Introduction

Platelet aggregation, the process by which platelets adhere to each other, has long been recognized as critical for hemostatic plug formation and thrombosis (1). Antiplatelet therapy that reduces platelet aggregation has become a central part of treatment to reduce cardiovascular and cerebrovascular disease incidence in at-risk patient groups (2). Clopidogrel is an irreversible antagonist for the platelet $P2Y_{12}$ adenosine diphosphate receptor, thereby inhibiting platelet function and reducing likelihood of thrombosis (3). In 2018 it was the 39th most commonly prescribed drug in the USA (20 million prescriptions) (4). Clopidogrel is a prodrug that requires transformation to its bioactive form by cytochrome P-450 (CYP) enzymes, primarily CYP2C19 (3). Loss-of-function (LoF) genetic polymorphisms in *CYP2C19* impair clopidogrel metabolism, with carriers of any LoF variant reported to have a 32.4% reduction in plasma active metabolite levels compared to non-carriers (3). Clopidogrel resistance (high on-treatment platelet reactivity) is predominantly caused by *CYP2C19* LoF variants, with a recent study of clopidogrel on-treatment reactivity reporting that 71.7% of LoF carriers show clopidogrel resistance, compared to 32.1% of non-carriers (5). *CYP2C19* LoF carriers therefore have decreased platelet inhibition with consequently increased risk of thrombosis (6).

LoF variants in the *CYP2C19* gene are common in many populations, with the *2 allele ranging from 15% frequency in Europeans (with ~27% of the population carrying at least one copy) to >30% in Asian populations (7). A recent 2017 meta-analysis of 15 studies (12 East Asian, 3 European ancestry predominantly from the USA) included 4,762 stroke or transient ischaemic attack (TIA) patients treated with clopidogrel; patients carrying any *CYP2C19* LoF alleles had nearly double the rates of incident strokes (Risk Ratio [RR] 1.92: 95% Confidence Intervals 1.10 to 2.06, p=0.01) compared to non-carriers (8). However, most studies thus far focused on specific hospitalized patient groups with short (up to 1 year) follow-ups. A 2011 systematic review concluded that a significant association with stent thrombosis was due to small study effect bias and replication diversity (9). Although the US Food and Drug Administration added a Boxed Warning to the clopidogrel label regarding poor metabolizers due to *CYP2C19* variants (10) in 2010, there is continued debate about whether the magnitude of effect of these variants on clinical outcomes justifies *CYP2C19* genotype (6) guided prescription or use of alternative medications unaffected by these variants. In 2019 a consensus statement from experts in the field recommended genotyping as an optional tool for guiding treatment in certain scenarios (11).

For peer review only - http://bmjopen.bmj.com/site/about/guidelines.xhtml

Given the widespread continuing use of clopidogrel, and the current scarcity of evidence on clinical outcomes due to the LoF variants in the primary care setting, we aimed to estimate the association between *CYP2C19* LoF alleles and incident hospital-diagnosed ischaemic stroke and MI in UK Biobank participants on treatment with clopidogrel. We also aimed to estimate longer-term (>1 year) outcomes, as these have previously been understudied, and to estimate outcomes of non-*CYP2C19* variants reported to have similar effects (12).

For peer review only

For peer review only - http://bmjopen.bmj.com/site/about/guidelines.xhtml

Methods

UK Biobank cohort

The UK Biobank recruited 503,325 community-based volunteers aged 40-70 years who visited one of 22 assessment centers in England, Scotland or Wales between 2006 and 2010 (13). Data collected at the baseline assessment included extensive questionnaires on demographic, health, and lifestyle information. Anthropometric measures were also taken, in addition to blood samples for future biochemical and genetic analysis. The North West Multi-Centre Research Ethics Committee approved the collection and use of UK Biobank data (Research Ethics Committee reference 11/NW/0382). This research was conducted under UK Biobank application 14631 (PI: DM). UK Biobank volunteers tended to be healthier than the general population at baseline assessment (14), however this largely prospective analysis of linked primary care data is less affected by sample response patterns than baseline parameters.

Prescription data

General practice prescriptions data is currently available for 230,096 (45.7%) participants, with >57 million prescribing events recorded. UK Biobank obtained data from the three main primary care electronic clinical record data providers (England: TPP/Vision; Scotland and Wales: EMIS/Vision). The extract date differs between the supplier's computer systems, and we used the following censoring dates: June 2016 (England), April 2017 (Scotland), and September 2017 (Wales). Detailed description of the data extracted and limitations are available from UK Biobank (15). Data includes prescription dates, drug code (in clinical Read v2, British National Formulary (BNF), or Dictionary of Medicines and Devices format (depending on supplier), drug name, and quantity, where available. We identified all prescribing events for clopidogrel and aspirin. Details for drug names and clinical codes used are available in the Supplementary Information and Supplementary Table 1.

In the prescribing data we identified all records matching a clopidogrel code and for each patient determined the date first prescribed, date last prescribed, number of total prescriptions, and average number of prescriptions over the time period. We analyzed data for patients who received two or more prescriptions, with at least one clopidogrel prescription for each 2-month period between the dates first/last prescribed. This last criterion excluded participants with longer gaps between prescriptions, who may not have been taking clopidogrel for the entire study period, thus minimizing immortal time bias (16).

For peer review only

For peer review only - http://bmjopen.bmj.com/site/about/guidelines.xhtml

Participants never prescribed clopidogrel were used for sensitivity analysis of *CYP2C19* LoF variants and relevant vascular outcomes.

CYP2C19 **genetic variants**

Directly genotyped genetic variants (n=805,426) were available for 488,377 UK Biobank participants, based on two almost identical genotyping platforms sharing >95% of variants: the Affymetrix Axiom UKB array (in 438,427 participants) and the Affymetrix UKBiLEVE array (in 49,950 participants). After extensive quality control by the central UK Biobank team (17), genotype imputation was successful in 487,442 participants and increased the number of genetic variants to ~96 million (17). Our primary analysis included 451,367 participants (93%) identified as genetically European (identified by genetic clustering, as described previously (18)), to reduce bias in genetic studies from population stratification, which arises when different ancestral groups are analyzed together in genetic analyses (19). Secondary analysis in other ancestral groups was not possible due to low numbers in the clopidogrel patients (n=492 non-Europeans with sufficient clopidogrel data; this group included all other ancestries such as South or East Asian, African, and mixed).

We analyzed the *CYP2C19* genetic variants (*2-*8 and *17) with well-documented effects on clopidogrel metabolism in the literature (9) and in the PharmGKB database (12): three loss-of-function alleles (*2, *3 and *8; intermediate or poor metabolizers) and one gain-of-function allele (*17; rapid metabolizer) were either directly genotyped (*3 and *8) or imputed with high confidence (*2 and *17: >99.9% imputation confidence). *CYP2C19**4 was imputed with <80% imputation confidence (74.4%) so was excluded from analysis (*4 is very rare so this has minimal impact on the analysis: with 0.25% allele frequency reported in the GnomAD (7) database https://gnomad.broadinstitute.org/). *CYP2C19* *5, *6 and *7 were not available in the genotyping data, as they are extremely rare, especially in European populations (<0.1% allele frequency in GnomAD (7)). See Supplementary Table 2 for details on individual variants used.

Non-*CYP2C19* **genetic variants**

We also investigated non-*CYP2C19* genetic variants that affect clopidogrel from two sources: we searched the PharmGKB database (12) for variants classified as being supported by moderate or high clinical annotation levels of evidence, i.e. associations that replicated in subsequent studies, even if the original study was small. In addition to variants in *CYP2C19* (level 1 “high” levels of evidence) one variant in *CES1* has level 2B “moderate” evidence:

For peer review only - http://bmjopen.bmj.com/site/about/guidelines.xhtml

rs71647871 was directly genotyped on the microarray but is rare, with only 249 heterozygotes in the clopidogrel analysis group (no homozygotes).

Lastly, we investigated variants identified in a genome-wide association study of clopidogrel active metabolite levels (rs187941554 and rs80343429) (20). Both variants are available in the UK Biobank imputed genotype data: rs187941554 was imputed with high confidence (99.9%), however the overall rs80343429 imputation accuracy was 81.7% (n=3 individual participants with low-confidence genotype calls - imputed genotype dose >0.25 and <0.75 - were excluded from analysis).

Disease ascertainment

Incident ischaemic stroke and MI ascertainment from hospital admissions records (Hospital Episode Statistics, HES) were available up to 14 years follow-up after baseline assessment, covering the entire period up to the date of censoring of primary care prescribing data (HES in England up to 30 September 2020: data from Scotland and Wales censored to 31 August 2020 and 28 Feb 2018, respectively). Diagnosis of ischaemic stroke was ascertained using ICD-10 code I63*. Diagnosis of MI was ascertained using ICD-10 codes I21*; I22*; I23*; I24*; I25*.

For prevalent ascertainment of diagnoses at baseline of transient ischaemic attack (TIA), MI/angina, atrial fibrillation, hypertension or peripheral vascular disease we used primary care records. Diagnosis codes (read v2) were downloaded from the CALIBER phenotype library (https://portal.caliberresearch.org/disease-or-syndrome) (21). Corresponding CTV3 codes were identified using the UK Biobank “Clinical coding classification systems and maps” resource (https://biobank.ctsu.ox.ac.uk/crystal/refer.cgi?id=592).

Statistical analysis

Data are from multiple sources (demographic and genetic data from UK Biobank assessment, clopidogrel prescribing data from linked electronic primary care records, and stroke and MI outcomes from linked electronic hospital inpatient records). Patients with any missing data needed for analysis were excluded, with details shown in the results.

Estimates of associations between genotype and incident disease were obtained from Cox’s proportions hazards regression models, with adjustment for age at first prescription, sex, and the first ten principal components of genetic ancestry (to control for population substructure). Patients entered the model 7 days after the date of first prescription of clopidogrel; this was to

For peer review only - http://bmjopen.bmj.com/site/about/guidelines.xhtml

exclude the small number of events that occurred immediately after being prescribed clopidogrel, before it could reasonably have had a preventative effect. Seven days was chosen because the effect of clopidogrel on platelet aggregation was significantly different between *CYP2C19* genotype groups after 7-10 days in a study of 375 patients (22). Patients exited the model on the last known date of clopidogrel prescription. STATA (v15.1) software was used for analysis. We visually inspected Kaplan-Meier plots and used STATA function `estat phtest, detail` to generate Schoenfeld residuals to test for violations of the proportional-hazards assumption.

Population attributable fraction in this analysis is the estimated proportion of all cases that would not have occurred if no participants carried the clopidogrel modifying genetic variants. It was calculated by multiplying the proportion of incident ischaemic strokes in the genotype carriers by (1 – 1 / Hazard Ratio for ischaemic stroke) and similarly for MI (23). This method is preferred to simple observed vs. expected cases as it uses the estimate from the time-to-event model, which has been adjusted for covariates (23). Lifetable probabilities of incident outcomes were estimated from age 37 to 79 (the minimum and maximum ages the patients were first prescribed clopidogrel, respectively) by *CYP2C19* genotype, applying observed incidence rates in each age group to a notional cohort (where all participants in the analysis had reached the maximum age group), estimating cumulative incident case numbers, using STATA command `sts list`.

Patient and public involvement

Patients and participants were and are extensively involved in the UK Biobank study itself. No patients were involved in developing the research question or the outcomes tested. A lay abstract was reviewed by members of the Peninsula Public Engagement Group ("PenPEG") in Exeter, who provided positive feedback on the importance of the study from a patient perspective.

For peer review only - http://bmjopen.bmj.com/site/about/guidelines.xhtml

Results

7,483 UK Biobank participants of European ancestry met inclusion criteria, with at least two prescriptions of clopidogrel recorded in primary care data (see Figure 1 for cohort flowchart with detailed inclusion/exclusion criteria). The mean age at first prescription was 64.3 years (SD 7.2), and 2,563 were female (34.2%). The clopidogrel exposure period ranged from 2 months to 18 years (mean 2.59 years, SD 2.98). Cohort selection is described in Table 1. Patients carrying at least one *CYP2C19* LoF variant (*2-*8, intermediate/poor clopidogrel metabolizers) did not have significantly different likelihood of receiving clopidogrel compared to non-carriers (Odds Ratio 1.01: 95% Confidence Intervals 0.96 to 1.05, p=0.75) and had similar prevalence of cardiovascular co-morbidities prior to diagnosis (Table 1).

CYP2C19 LoF associations with incident ischaemic stroke

Incident hospital-diagnosed ischaemic stroke (cerebral infarction) was identified in 110 of 7,477 participants (1.47%) eligible for analysis (after excluding n=6 who had stroke within 7 days of first prescription). *CYP2C19* LoF (*2-*8, intermediate/poor metabolizers) carriers (n=2,144, 28.7%) were more likely to have an incident ischaemic stroke whilst on treatment for clopidogrel than patients without *CYP2C19* LoF genetic variants (Hazard Ratio (HR) 1.53: 95% CI 1.04 to 2.26, p=0.031). See Table 2 for details and Figure 2A for Kaplan-Meier plot. 1.29% of the patients without any *CYP2C19* LoF genetic variants had an incident stroke during the prescribing period (mean 2.59 years between first and last clopidogrel prescription, SD 2.98) compared to 1.91% of the *CYP2C19* LoF carriers: this absolute 0.64% excess is statistically significant (95% CI 0.021% to 1.35%, p=0.027).

To test whether the effect of *CYP2C19* LoF genotypes on ischaemic stroke risk was specific to clopidogrel, and not via a separate but unknown biological mechanism, we analyzed the 198,868 UK Biobank participants with GP prescribing data in whom clopidogrel was never prescribed (see Figure 1). There were 512 incident ischaemic stroke events in this group after baseline assessment and before the end of Feb 2016 (earliest censoring date for GP prescribing data). *CYP2C19* LoF carriers were not at significantly increased risk of ischaemic strokes compared to non-carriers (HR 1.12: 95% CI 0.93 to 1.35, p=0.24).

To calculate the population attributable fraction (PAF) we multiplied the proportion of incident ischaemic strokes that occurred in that patients who had intermediate or low metabolizer variants (41/110) by (1-1/HR for ischaemic stroke = 1-1/1.53 = 0.346) giving 0.129 (see Methods). We therefore estimate the PAF for ischaemic stroke - the proportion of ischaemic strokes in this population that would not have occurred if clopidogrel efficacy was not affected by *CYP2C19* LoF variants - to be 12.9% (95% CI 1.4% to 20.8%). Kaplan-Meier curves for

For peer review only - http://bmjopen.bmj.com/site/about/guidelines.xhtml

diagnosis of incident ischaemic strokes in participants prescribed clopidogrel are shown in Figure 2. In lifetable estimates based on observed incidence rates from 36 to 79 (the minimum and maximum ages at which clopidogrel was first prescribed) the risk of ischaemic stroke in CYP2C19 LoF carriers was 22.5% (95% CI 14.4% to 34.0%), compared with 15.4% (95% CI 11.4% to 20.5%) in non-carriers.

We performed secondary analyses estimating outcomes for participants prescribed clopidogrel for >1 year (n=4,316: 57.8%). *CYP2C19* LoF carriers had significantly increased risk of incident strokes (HR 1.63: 95% CI 1.01 to 2.64, p=0.047) in this period (Table 2). However the association with only those events within the first year of prescription was not statistically significant (HR 1.33: 95% CI 0.69 to 2.57, p=0.390). In tests of the proportional-hazards assumption (see Methods) in the model using all available events there was no evidence that the hazard ratio changed over time (p=0.42). The analysis using all participants suggests LoF carriers are at increased risk for the duration of the clopidogrel prescribing (HR 1.53: 95% CI 1.04 to 2.26, p=0.031).

In sensitivity analysis we adjusted the main analysis of *CYP2C19* LoF carriers for history of stroke, TIA, MI/angina, PAD, and atrial fibrillation to assess whether disease indication modified the effect of genotype on ischaemic stroke risk and found the estimate to be unaffected (HR 1.56: 95% CI 1.06 to 2.31, p=0.024).

We investigated hemorrhagic strokes (ICD-10 codes I61* and I62*), however there were too few diagnoses made (n=9 in the 7,483 participants included in the study) during the clopidogrel prescribing period, so no analysis was performed.

We also analyzed outcomes for the *17 (gain of function) genotype carriers (rapid metabolizers). *17 carriers were not at altered risk of incident stroke during the prescribing period (HR 0.98: 95% CI 0.61 to 1.57, p=0.930, Supplementary Table 3) compared to *1/*1 patients.

CYP2C19 **LoF associations with incident myocardial infarction**

Incident hospital-diagnosed myocardial infarctions (MI) occurred in 1,823 of 7,359 participants (24.8%), after excluding 124 subjects who had MIs within 7 days of first clopidogrel prescription. *CYP2C19* LoF (*2-*8, intermediate/poor metabolizers) carriers (n=2,100, 28.5%) were more likely to have an incident MI whilst being prescribed clopidogrel than patients without *CYP2C19* LoF genetic variants (HR 1.14: 95% CI 1.04 to 1.26, p=0.008, see Table 2 for details and Figure 2B for Kaplan-Meier plot). The PAF for MI was 3.73% (95% CI 1.2% to 6.3%).

For peer review only - http://bmjopen.bmj.com/site/about/guidelines.xhtml

We performed secondary analysis investigating the participants prescribed clopidogrel for >1 year (n=3,511: 47.7%). *CYP2C19* LoF carriers were not at significantly increased risk of incident MIs (HR 1.09: 95% CI 0.90 to 1.33, p=0.37) in this period (Table 2). However, LoF carriers did have significantly increased risk of MI within the first year of clopidogrel prescription (HR 1.16: 95% CI 1.03 to 1.31, p=0.011). In tests of the proportional-hazards assumption (see Methods) in the model using all available events there was no evidence that the hazard ratio changed over time (p=0.70). The analysis using all participants suggests LoF carriers are at increased risk for the duration of the clopidogrel prescribing (HR 1.14: 95% CI 1.04 to 1.26, p=0.008).

We also analyzed the *17 (gain of function) genotype carriers (rapid metabolizers). *17 carriers did not have significantly different risk of incident MI during the prescribing period (HR 0.95: 95% CI 0.85 to 1.06, p=0.33, Supplementary Table 3) compared to *1/*1 patients.

The association between *CYP2C19* LoF carriers and MI in participants never prescribed clopidogrel (n=4,390 MIs) was not significant (HR 1.03: 95% CI 0.96 to 1.10, p=0.41).

In secondary analysis of any ischaemic event (stroke or MI, *n*=1,886 events in 7,354 patients included in analysis) *CYP2C19* LoF (*2-*8, intermediate/poor metabolizers) carriers were more likely to have an event whilst being prescribed clopidogrel than patients without *CYP2C19* LoF genetic variants (HR 1.17: 95% Cis 1.06 to 1.29, p=0.002).

Dual antiplatelet therapy: clopidogrel co-prescribing with aspirin

Of 7,483 patients prescribed clopidogrel 3,730 (49.9%) were also prescribed aspirin at least once during the analysis period. *CYP2C19* LoF carriers had slightly higher likelihood of ever receiving a prescription of aspirin compared to non-carriers during their clopidogrel exposure period (n=1,118 (52.2%) vs. n=2,629 (49.3%), odds ratio (OR) from logistic regression 1.14: 95% CI 1.02 to 1.26, p=0.017). Ever being prescribed aspirin did not significantly interact with *CYP2C19* LoF carrier status in the model of incident stroke or MI (p>0.05). The association between *CYP2C19* LoF carrier status and incident stroke or MI was consistent (albeit attenuated due reduced sample sizes in subgroups) in participants who received an aspirin prescription (n=3,730. Stroke HR 1.61: 95% CI 0.87 to 2.98, p=0.13; MI HR 1.10; 95% CI 0.98 to 1.24, p=0.10) and those who did not (n=3,753. Stroke HR 1.48: 95% CI 0.89 to 2.46, p=0.13; MI HR 1.19: 95% CI 0.98 to 1.45, p=0.087).

For peer review only - http://bmjopen.bmj.com/site/about/guidelines.xhtml

Analysis of non-*CYP2C19* genetic variation associated with clopidogrel active metabolite levels

A rare variant in *CES1* (rs71647871, n=249 CT heterozygotes) with moderate clinical annotation evidence for clopidogrel on the PharmGKB database (12) was not significantly associated with strokes or MIs (stroke HR 0.86: 95% CI 0.27 to 2.70, p=0.79; MI HR 1.26: 95% CI 0.99 to 1.60, p=0.057), although the MI association was borderline.

Two genetic variants identified in a GWAS of clopidogrel active metabolite levels (20) were also analyzed. Despite its low frequency in the population, rs187941554 (n=32 GA heterozygotes, 0.4% of 7,468) was associated with substantially raised risk of incident stroke (n strokes=2; HR 5.44: 95% CI 1.33 to 22.30, p=0.019) but not MIs (HR 1.20: 95% CI 0.62 to 2.32, p=0.58). The rs80343429 A allele was not associated with increased risk of stroke or MI (stroke HR 1.19: 95% CI 0.68 to 2.09, p=0.53; MI HR 1.09: 95% CI 0.94 to 1.26, p=0.25).

For peer review only - http://bmjopen.bmj.com/site/about/guidelines.xhtml

Discussion

Clopidogrel requires metabolic activation to be effective, mainly by the CYP2C19 liver enzyme. *CYP2C19* LoF genetic variants (implying intermediate or low metabolizer status) have been shown to increase stroke and MI rates mainly in hospitalized patients with acute ischaemic conditions (or undergoing related interventions such as cardiac stents (24)), with little data on outcomes in primary care or over longer follow-up periods. In this study of 7,483 UK Biobank primary care patients prescribed clopidogrel, carriers of *CYP2C19* LoF alleles had substantially increased risks of incident ischaemic strokes and myocardial infarction. We also showed that carriers of LoF alleles are at increased risk of stroke even during longer-term (>1 year) therapy; the effect is not limited to acute stroke patients.

Our results are consistent with previous studies of clopidogrel efficacy in *CYP2C19* LoF genotype carriers, although the previous evidence was predominantly from high-risk acutely hospitalized patient groups (25–28), with limited follow-up periods (rarely over 12 months). Some studies reported that the effect of *CYP2C19* genotype on reducing clopidogrel efficacy was limited to the short-term (1-6 months) only (29,30), but in our community based longer term follow-up we found no evidence for deviation from the proportional hazards assumptions, with survival analysis results suggesting sustained effects over the follow-up period. We had limited sample size and therefore limited statistical power to estimate effects for specific periods, but the intermediate or low metabolizer variant association with excess incident strokes was significant in the subgroup prescribed clopidogrel for more than one year.

A recent 2019 meta-analysis of 16 randomized clinical trials (31) found that dual antiplatelet therapy with aspirin was associated with significantly lower stroke rates (RR 0.80: 0.72 to 0.89), however some evidence of increased major bleeding was also observed (RR 1.90: 1.33 to 2.72). The authors noted that common *CYP2C19* LoF variants may account for the effectiveness of dual therapy over clopidogrel monotherapy (31). In our study we found LoF carriers still had increased stroke or MI risk, even if co-prescribed aspirin.

Clopidogrel is a recommended antiplatelet medication for the prevention of stroke in the USA (2) and is the currently recommended antiplatelet medication for prevention of stroke or TIA in the UK (32). However, antiplatelet medications such as ticagrelor and prasugrel are not dependent on CYP2C19 metabolism and reportedly have better clinical outcomes in LoF carriers compared to clopidogrel (33). A recent meta-analysis of 16 studies (8 RCTs and 8 cohorts) concluded that genotype-guided antiplatelet treatment (primarily based on *CYP2C19* LoF alleles) improved patient outcomes for major cardiovascular events (e.g. Relative Risk for MI 0.45: 95% CI 0.35 to 0.58, p<0.00001), with decreased risk of major bleeding (28). Treatment strategy in the different metabolizer groups varied between individual studies, but

For peer review only - http://bmjopen.bmj.com/site/about/guidelines.xhtml

included use of higher doses of clopidogrel (150 mg/d instead of the usual 75 mg/d), or prescribing of prasugrel or ticagrelor, or a combination. Other recent studies in different populations, especially in patients with acute coronary syndrome, also support this conclusion (25–27). Therefore, further work is needed in primary care populations to inform optimal antiplatelet care, given our estimate that over 12% of incident strokes in the UK study population on clopidogrel could be avoided if low and intermediate metabolizers could achieve the same clinical outcomes as are being achieved in the normal metabolizer group. Economic analysis in the UK comparing clopidogrel (out of patent at the time of writing) to alternatives not necessarily out of patent are needed.

This is the largest single study of primary care patients prescribed clopidogrel, incorporating *CYP2C29* genetic data and longer (>1 year) follow-up of clinical outcomes. However, this study is not without limitations. UK Biobank participants tended to be healthier than the general population at baseline assessment (14), however, linked primary care data used follow-up analysis should be unaffected by sample response patterns. Population attributable risk may be underestimated, as UK Biobank has a limited age range (mean age of 64 years at first prescription, max 79) that does not include many older people at high risk of strokes or MIs, so estimates of absolute risk may be greater in the general population. Further work is required to model this in other countries. The availability of only primary care prescribing (with no hospital prescribing details available) may mean that some clopidogrel prescriptions have been missed and therefore some patients or exposure periods excluded, but likely only for periods during and soon after hospital admissions. Although this is a relatively large study of 7,483 patients, stratified analyses (such as *2 heterozygous/homozygous groups, or by aspirin co-prescribing) were underpowered. The analysis was restricted to UK Biobank participants of genetically European ancestry (93% of the cohort); only 492 non-European participants had sufficient clopidogrel data, but this included all other ancestries including South or East Asian, African, and mixed, so analysis was not possible. Future analyses with more data will be needed to address these research questions. Similarly, analyses of the low frequency variants in larger samples may be needed to refine estimates, including for rs187941554, for which our nominally significant association (HR 5.44: 95% CIs 1.33 to 22.30, p=0.019) was based on only two incident strokes in 32 subjects.

To conclude, intermediate and low metabolizers (due to *CYP2C19* loss of function alleles) who were prescribed clopidogrel in the primary care setting had substantially increased risks of incident hospital-diagnosed strokes and MIs, compared to normal metabolizers. Evidence for improved patient outcomes with *CYP2C19* genotype-guided antiplatelet therapy is increasing, especially in the acute setting. Genotype guided section of antiplatelet medications may improve outcomes for primary care patients carrying *CYP2C19* variants.

For peer review only - http://bmjopen.bmj.com/site/about/guidelines.xhtml

Acknowledgements

Access to UK Biobank resource was granted under Application Number 14631. We would like to thank UK Biobank participants and coordinators for this dataset. The authors would like to acknowledge the use of the University of Exeter High-Performance Computing (HPC) facility in carrying out this work.

Author Contributions

LCP performed the analysis, interpreted results, created the figures, searched literature, and co-wrote the manuscript. DT and HF performed the literature search, performed analyses, and contributed to the manuscript. JA and JD performed analyses and contributed to the manuscript. CK, GAK, LF and JB contributed to data interpretation and contributed to the manuscript. JM provided expert clinical interpretation of the data and contributed to the manuscript. DM oversaw data analysis, interpretation, and literature searching, and led the writing of the manuscript. DM is the guarantor. The corresponding author attests that all listed authors meet authorship criteria and that no others meeting the criteria have been omitted.

For peer review only

Funding

LCP and DM are supported by the University of Exeter Medical School and the University of Connecticut School of Medicine. DT is funded by the Ministry of National Education, Republic of Turkey. JA was supported by an award to DM by the UK Medical Research Council (MR/S009892/1). JD is also supported by the Alzheimer's Society [grant: 338 (AS-JF-16b-007)]. GAK is supported by the Travelers Chair in Geriatrics and Gerontology. JB is funded by an Expanding Excellence in England (E3) research grant awarded to the University of Exeter. LF is supported by the National Institute on Aging, NIH. JM is funded by a National Institute for Health Research Fellowship(NIHR301445). This publication presents independent research funded by the National Institute for Health Research (NIHR). The views expressed are those of the author(s) and not necessarily those of the NHS, the NIHR or the Department of Health and Social Care. The funders had no input in the study design; in the collection, analysis, and interpretation of data; in the writing of the report; or in the decision to submit the article for publication. The researchers acted independently from the study sponsors in all aspects of this study.

Competing interests

All authors have completed the ICMJE uniform disclosure form (at www.icmje.org/coi_disclosure.pdf) and declare: no support from any organisation for the submitted work; no financial relationships with any organisations that might have an interest

For peer review only - http://bmjopen.bmj.com/site/about/guidelines.xhtml

in the submitted work in the previous three years; no other relationships or activities that could appear to have influenced the submitted work.

Ethical approval

The North West Multi-Centre Research Ethics Committee approved the collection and use of UK Biobank data (Research Ethics Committee reference 11/NW/0382).

Data sharing

Data are available on application to the UK Biobank (www.ukbiobank.ac.uk/register-apply). Additional data are available from the corresponding author on reasonable request.

Transparency

The lead author (DM) affirms that this manuscript is an honest, accurate, and transparent account of the study being reported; that no important aspects of the study have been omitted; and that any discrepancies from the study as planned (and, if relevant, registered) have been explained.

Supplementary Material

Supplementary Methods

Supplementary Tables 1-3

For peer review only - http://bmjopen.bmj.com/site/about/guidelines.xhtml

References

1. Jackson S. The growing complexity of platelet aggregation. Blood [Internet]. 2007;109(12):5087–95.

2. Powers WJ, Rabinstein AA, Ackerson T, Adeoye OM, Bambakidis NC, Becker K, et al. 2018 Guidelines for the Early Management of Patients With Acute Ischemic Stroke: A Guideline for Healthcare Professionals From the American Heart Association/American Stroke Association. Stroke. 2018;49(3):e46–110.

3. Mega JL, Close SL, Wiviott SD, Shen L, Hockett RD, Brandt JT, et al. Cytochrome P-450 Polymorphisms and Response to Clopidogrel. N Engl J Med [Internet]. 2009 Jan 22 [cited 2020 Jul 8];360(4):354–62.

4. Kane SP. ClinCalc DrugStats: Clopidogrel Bisulphate [Internet]. 2021 [cited 2021 Jan 20].

5. Fu H, Hu P, Ma C, Peng F, He Z. Association of clopidogrel high on-treatment reactivity with clinical outcomes and gene polymorphism in acute ischemic stroke patients. Medicine (Baltimore) [Internet]. 2020 Apr;99(15):e19472.

6. Simon T, Danchin N. Clinical Impact of Pharmacogenomics of Clopidogrel in Stroke. Circulation [Internet]. 2017 Jan 3;135(1):34–7.

7. Karczewski KJ, Francioli LC, Tiao G, Cummings BB, Alföldi J, Wang Q, et al. The mutational constraint spectrum quantified from variation in 141,456 humans. Nature. 2020;581(7809):434–43.

8. Pan Y, Chen W, Xu Y, Yi X, Han Y, Yang Q, et al. Genetic Polymorphisms and Clopidogrel Efficacy for Acute Ischemic Stroke or Transient Ischemic Attack. Circulation [Internet]. 2017 Jan 3;135(1):21–33.

9. Bauer T, Bouman HJ, Van Werkum JW, Ford NF, Ten Berg JM, Taubert D. Impact of CYP2C19 variant genotypes on clinical efficacy of antiplatelet treatment with clopidogrel: Systematic review and meta-analysis. BMJ. 2011;343(7819):1–18.

10. U.S. Food & Drug Administration. FDA Drug Safety Communication: Reduced effectiveness of Plavix (clopidogrel) in patients who are poor metabolizers of the drug [Internet]. 2010 [cited 2021 Mar 29].

11. Sibbing D, Aradi D, Alexopoulos D, ten Berg J, Bhatt DL, Bonello L, et al. Updated Expert Consensus Statement on Platelet Function and Genetic Testing for Guiding P2Y12 Receptor Inhibitor Treatment in Percutaneous Coronary Intervention. JACC

For peer review only - http://bmjopen.bmj.com/site/about/guidelines.xhtml

Cardiovasc Interv [Internet]. 2019 Aug;12(16):1521–37.

12. Whirl-Carrillo M, McDonagh EM, Hebert JM, Gong L, Sangkuhl K, Thorn CF, et al. Pharmacogenomics Knowledge for Personalized Medicine. Clin Pharmacol Ther [Internet]. 2012 Oct;92(4):414–7.

13. Sudlow C, Gallacher J, Allen N, Beral V, Burton P, Danesh J, et al. UK Biobank: An Open Access Resource for Identifying the Causes of a Wide Range of Complex Diseases of Middle and Old Age. PLoS Med. 2015;12(3):e1001779.

14. Fry A, Littlejohns TJ, Sudlow C, Doherty N, Adamska L, Sprosen T, et al. Comparison of Sociodemographic and Health-Related Characteristics of UK Biobank Participants With Those of the General Population. Am J Epidemiol. 2017 Nov 1;186(9):1026–34.

15. UK Biobank. UK Biobank Primary Care Linked Data Version 1.0 [Internet]. 2019 [cited 2019 Sep 30].

16. Yadav K, Lewis RJ. Immortal Time Bias in Observational Studies. JAMA [Internet]. 2021 Feb 16;325(7):686.

17. Bycroft C, Freeman C, Petkova D, Band G, Elliott LT, Sharp K, et al. The UK Biobank resource with deep phenotyping and genomic data. Nature. 2018;562(7726):203–9.

18. Thompson WD, Tyrrell J, Borges M-C, Beaumont RN, Knight BA, Wood AR, et al. Association of maternal circulating 25(OH)D and calcium with birth weight: A mendelian randomisation analysis. PLoS Med [Internet]. 2019 Jun;16(6):e1002828.

19. Hellwege JN, Keaton JM, Giri A, Gao X, Velez Edwards DR, Edwards TL. Population Stratification in Genetic Association Studies. Curr Protoc Hum Genet [Internet]. 2017 Oct 13;95(1).

20. Backman JD, O’Connell JR, Tanner K, Peer CJ, Figg WD, Spencer SD, et al. Genome-wide analysis of clopidogrel active metabolite levels identifies novel variants that influence antiplatelet response. Pharmacogenet Genomics [Internet]. 2017 Apr [cited 2020 Jul 1];27(4):159–63.

21. Kuan V, Denaxas S, Gonzalez-Izquierdo A, Direk K, Bhatti O, Husain S, et al. A chronological map of 308 physical and mental health conditions from 4 million individuals in the English National Health Service. Lancet Digit Heal [Internet]. 2019 Jun;1(2):e63–77.

22. Yi X, Lin J, Wang Y, Zhou Q, Wang C, Cheng W, et al. Association of cytochrome P450 genetic variants with clopidogrel resistance and outcomes in acute ischemic

For peer review only - http://bmjopen.bmj.com/site/about/guidelines.xhtml

stroke. J Atheroscler Thromb. 2016;23(10):1188–200.

23. Mansournia MA, Altman DG. Population attributable fraction. BMJ [Internet]. 2018 Feb 22;k757.

24. Pereira NL, Rihal C, Lennon R, Marcus G, Shrivastava S, Bell MR, et al. Effect of CYP2C19 Genotype on Ischemic Outcomes During Oral P2Y12 Inhibitor Therapy. JACC Cardiovasc Interv [Internet]. 2021 Mar;1–12.

25. Ramesh S, Socrates S, Rajasekaran MA, Senguttuvan N. Incidence of major adverse cardiovascular events with genotype test guided antiplatelet treatment strategy after percutaneous coronary intervention. Indian Heart J [Internet]. 2020 Nov;72(6):589–92.

26. Zhang M, Wang J, Zhang Y, Zhang P, Chao Y, Gao M, et al. Effects of individualized antiplatelet therapy, based on CYP2C19 genotyping, on platelet function in patients underwent percutaneous coronary intervention. Perfusion [Internet]. 2020 Dec 10;026765912097858.

27. Russmann S, Rahmany A, Niedrig D, Hatz K-D, Ludin K, Burden AM, et al. Implementation and management outcomes of pharmacogenetic CYP2C19 testing for clopidogrel therapy in clinical practice. Eur J Clin Pharmacol [Internet]. 2020 Nov 26;

28. Zhang H, Xiang Q, Liu Z, Mu G, Xie Q, Zhou S, et al. Genotype-guided antiplatelet treatment versus conventional therapy: A systematic review and meta-analysis . Br J Clin Pharmacol. 2020;(October):1–17.

29. Wang Y, Cai H, Zhou G, Zhang Z, Liu X. Effect of CYP2C19*2 and *3 on clinical outcome in ischemic stroke patients treated with clopidogrel. J Neurol Sci [Internet]. 2016 Oct;369:216–9.

30. G. C, G. P, P. F, B. L, M. T, M. M, et al. Prospective evaluation of on-clopidogrel platelet reactivity over time in patients treated with percutaneous coronary intervention: Relationship with gene polymorphisms and clinical outcome. J Am Coll Cardiol [Internet]. 2011;57(25):2474–83.

31. Kheiri B, Osman M, Abdalla A, Haykal T, Swaid B, Ahmed S, et al. Clopidogrel and aspirin after ischemic stroke or transient ischemic attack: an updated systematic review and meta-analysis of randomized clinical trials. J Thromb Thrombolysis [Internet]. 2019;47(2):233–47.

32. NICE. NICE (UK National Institute for Health and Care Excellence) guidelines - antiplatelet treatment (September 2018 guidance) [Internet]. 2018 [cited 2020 Jun 26].

For peer review only - http://bmjopen.bmj.com/site/about/guidelines.xhtml

33. Yoon HY, Lee N, Seong J, Gwak HS. Efficacy and safety of clopidogrel versus prasugrel and ticagrelor for coronary artery disease treatment in patients with CYP2C19 LoF alleles: a systemic review and meta-analysis. Br J Clin Pharmacol [Internet]. 2020 Aug 28;86(8):1489–98.

For peer review only

For peer review only - http://bmjopen.bmj.com/site/about/guidelines.xhtml

Figure Legends

Figure 1: UK Biobank participants eligible for analysis: cohort flowchart

Cohort flowchart shows how the eligible participants to study were identified. Summary statistics for the 7,483 participants are available in Table 1. *Participants never prescribed clopidogrel were used for sensitivity analysis of CYP2C19 LoF variants and vascular outcomes.

Figure 2: Kaplan-Meier plots of strokes and MIs in patients prescribed clopidogrel, stratified by CYP2C19 Loss of Function genotypes

Kaplan-Meier failure plots for A) incident ischaemic stroke and B) incident MI showing cumulative hazard (%) with increasing time taking clopidogrel in patients prescribed clopidogrel for at least 2 months, stratified by CYP2C19 genotype (carriers of any *2-*8 Loss of Function variant, “intermediate/poor metabolizers of clopidogrel,” vs. *1/*1 normal metabolizers).

For peer review only

For peer review only - http://bmjopen.bmj.com/site/about/guidelines.xhtml

1 **Table 1: Summary of UK Biobank participants with GP-prescribed clopidogrel**

	CYP2C19 genotype	
	**Normal metabolizer (*1/*1)**	**Intermediate/ poor (any *2-*8)**
Number of participants (% of total n=7,483)	5,338 (71.3)	2,145 (28.7)
Females, n (% of genotype group)	1,847 (34.6)	712 (33.2)
Age at first clopidogrel prescription		
Minimum : maximum	36.5 to 78.8	37.6 to 78.1
Mean (SD)	64.1 (7.3)	64.3 (7.2)
Diagnoses* prior to clopidogrel prescription, n (%)		
Ischaemic stroke	509 (9.5)	240 (11.2)
Transient ischaemic attack	1,142 (21.4)	422 (19.7)
Myocardial infarction / angina	2,980 (55.8)	1,267 (59.1)
Atrial fibrillation	408 (7.6)	180 (8.4)
Hypertension	3,072 (57.6)	1,277 (59.5)
Peripheral vascular disease	442 (8.3)	177 (8.3)
Any of the above six diagnoses	4,863 (91.1)	1,982 (92.4)
Number of clopidogrel prescriptions		
Minimum : maximum	2 to 467.0	2 to 494.0
Mean (SD)	28.3 (36.7)	27.9 (36.9)
Years between first and last clopidogrel prescription		
Minimum : maximum	0.2 to 17.9	0.2 to 17.3
Mean (SD)	2.6 (3.0)	2.5 (2.9)
Also prescribed aspirin whilst taking clopidogrel, n (%)	2,632 (49.3)	1,119 (52.2)
Ischaemic stroke whilst taking clopidogrel, n (%)	69 (1.3)	41 (1.9)
Myocardial infarction whilst taking clopidogrel, n (%)	1,268 (24.1)	554 (26.4)

*Analysis of European-ancestry participants with >1 clopidogrel prescription in the available GP prescribing data. Participants excluded if clopidogrel prescribing frequency was less than once every 2 months. Events occurring after the last known date of clopidogrel prescription are also excluded. * Ischaemic stroke diagnoses are from hospital in-patient records only; all other disease also include diagnoses recorded in primary care.*

2

For peer review only - http://bmjopen.bmj.com/site/about/guidelines.xhtml

Table 2: *CYP2C19* genotype associations with incident ischaemic stroke and MI events in patients prescribed clopidogrel

Outcome, model	*CYP2C19 genotype*	N	N cases	Person-years	HR	95% CIs		*p*
**Ischaemic stroke**								
All participants	Normal metabolizer (*1/*1)	5,333	69	13,248				
	Intermediate/poor (any *2-*8)	2,144	41	5,110	1.53	1.04	2.26	0.031
	Total	*7,477*	*110*	*18,358*				
Events in first year only	Normal metabolizer (*1/*1)	5,333	25	4,419				
	Intermediate/poor (any *2-*8)	2,144	14	1,789	1.33	0.69	2.57	0.390
	Total	*7,477*	*39*	*6,208*				
Events after first year only	Normal metabolizer (*1/*1)	3,071	44	8,829				
	Intermediate/poor (any *2-*8)	1,245	27	3,322	1.63	1.01	2.64	0.047
	Total	*4,316*	*71*	*12,151*				
**Myocardial Infarction**								
All participants	Normal metabolizer (*1/*1)	5,259	1,269	10,093				
	Intermediate/poor (any *2-*8)	2,100	554	3,709	1.14	1.04	1.26	0.008
	Total	*7,359*	*1,823*	*13,803*				
Events in first year only	Normal metabolizer (*1/*1)	5,255	906	3,831				
	Intermediate/poor (any *2-*8)	2,099	415	1,511	1.16	1.03	1.31	0.011
	Total	*7,354*	*1,321*	*5,342*				
Events after first year only	Normal metabolizer (*1/*1)	2,529	362	6,258				
	Intermediate/poor (any *2-*8)	982	139	2,198	1.09	0.90	1.33	0.370
	Total	*3,511*	*501*	*8,455*				

Analysis of European-ancestry participants with >1 clopidogrel prescription in the available GP prescribing data. Participants excluded if clopidogrel prescribing frequency was less than once every 2 months. Events <1 week after first clopidogrel prescription are excluded. Events occurring after the last known date of clopidogrel prescription are also excluded. HR=Hazard Ratios from Cox's proportional hazards regression models adjusted for age at first clopidogrel prescription, sex, and genetic principal components of ancestry 1-10. CI = Confidence Intervals.

For peer review only

1

For peer review only

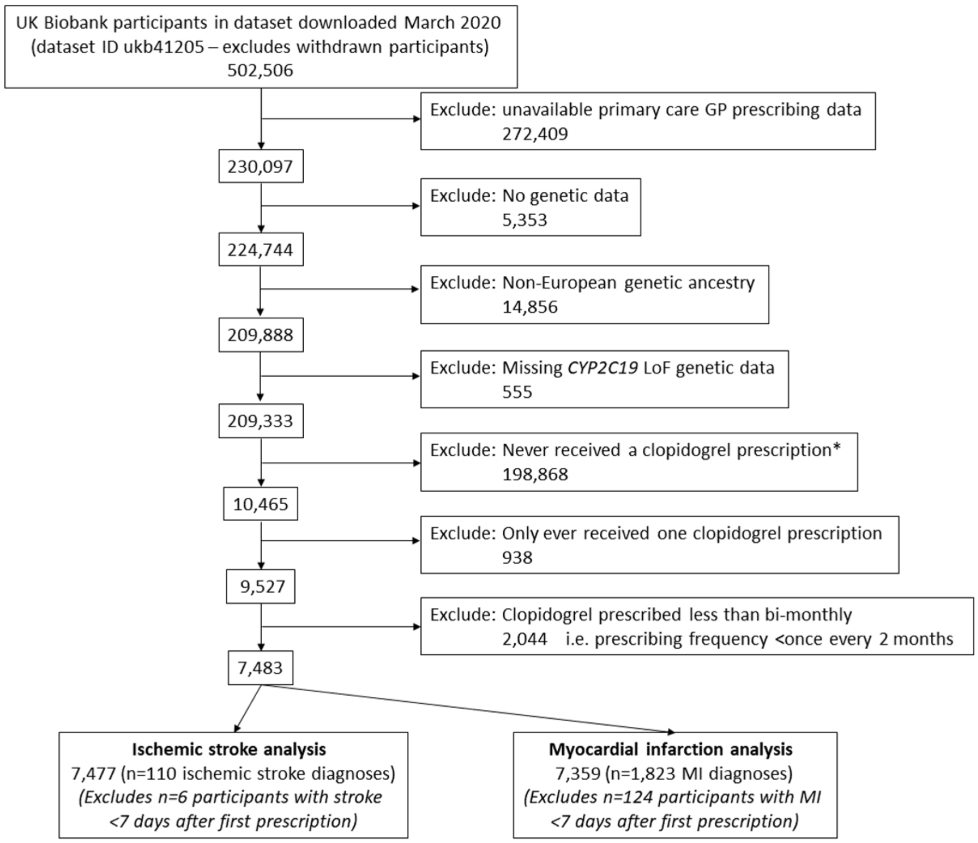

Figure 1: UK Biobank participants eligible for analysis: cohort flowchart
Cohort flowchart shows how the eligible participants to study were identified. Summary statistics for the 7,483 participants are available in Table 1. *Participants never prescribed clopidogrel were used for sensitivity analysis of CYP2C19 LoF variants and vascular outcomes.

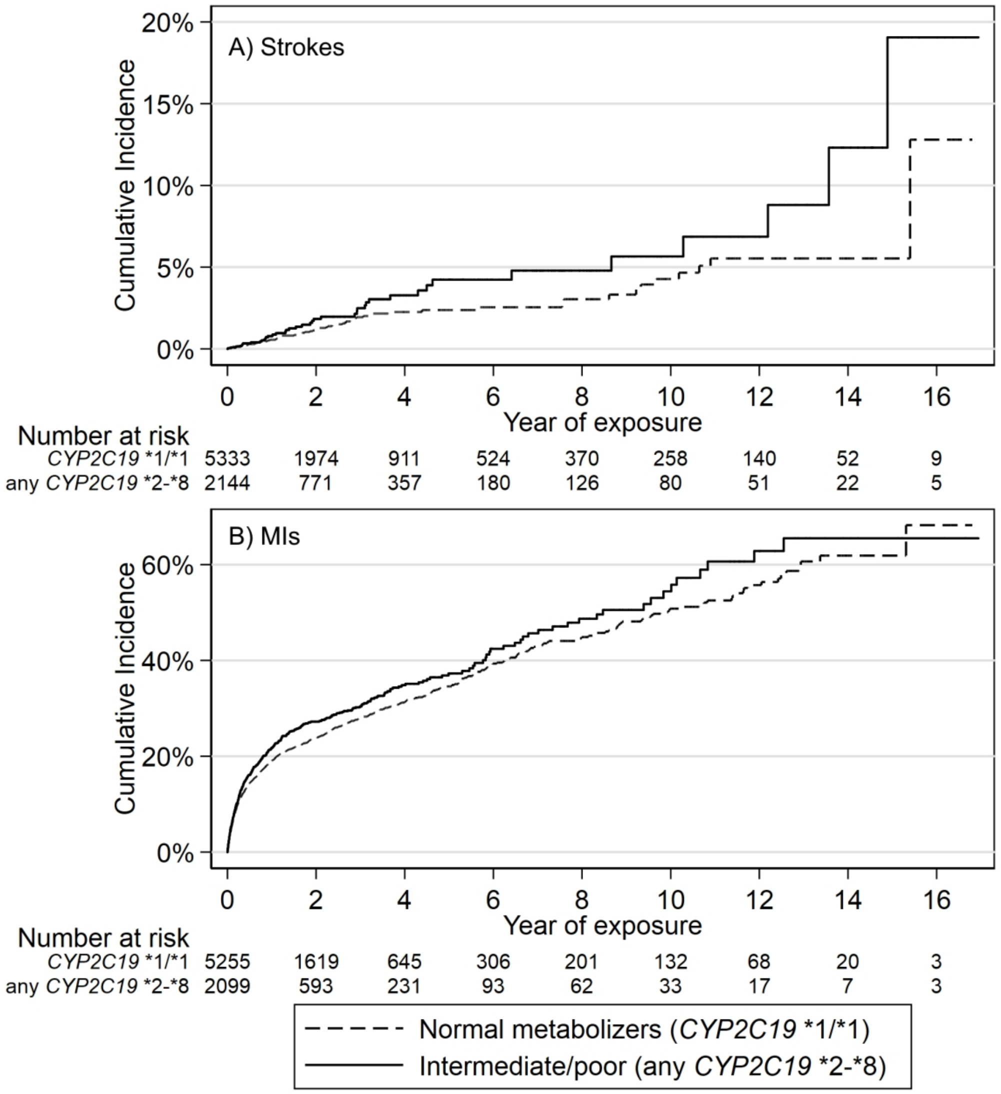

Figure 2: Kaplan-Meier plots of strokes and MIs in patients prescribed clopidogrel, stratified by CYP2C19 Loss of Function genotypes

Kaplan-Meier failure plots for A) incident ischaemic stroke and B) incident MI showing cumulative hazard (%) with increasing time taking clopidogrel in patients prescribed clopidogrel for at least 2 months, stratified by CYP2C19 genotype (carriers of any *2-*8 Loss of Function variant, "intermediate/poor metabolizers of clopidogrel," vs. *1/*1 normal metabolizers).

SUPPLEMENTARY MATERIAL

Genetic variation in activating clopidogrel: long-term outcomes in a large community cohort

Pilling et al.

Contents

Supplementary Methods 1
Prescription data 1
Supplementary Table 1: prescription codes in UK Biobank 2
Supplementary Table 2: *CYP2C19* genotypes in UK Biobank 3
Supplementary Table 3: *CYP2C19* associations with incident outcomes stratified by *17 genotype 4

Supplementary Methods

Prescription data

To identify prescriptions of clopidogrel and aspirin we used the UK Biobank "Coding system lookups and mappings" file (https://biobank.ctsu.ox.ac.uk/crystal/refer.cgi?id=592). For clopidogrel we searched for "clopidogrel|plavix|grepid" to include other relevant drug names. For aspirin we searched for "aspirin|nu-seals|caprin|disprin" also including relevant alternate names. We then used the associated read 2 codes and drug names in the `gp_scripts` UK Biobank prescription data for the participants. We did not use BNF codes as the resolution was not great enough to uniquely identify individual drugs.

For peer review only - http://bmjopen.bmj.com/site/about/guidelines.xhtml

Supplementary Table 1: prescription codes in UK Biobank

medication	term_description	read_2	bnf_code
clopidogrel	clopidogrel	bu5..	02.09.00.00
	clopidogrel 300mg tablets	bu54.	02.09.00.00
	clopidogrel 75mg tablets	bu51.	02.09.00.00
	grepid 75mg tablets	bu55.	02.09.00.00
	plavix 300mg tablets	bu53.	02.09.00.00
	plavix 75mg tablets	bu52.	02.09.00.00
aspirin	*aspirin 100mg m/r tablets	bu29.	02.09.00.00
	*aspirin 300mg m/r tablets	bu2b.	02.09.00.00
	*aspirin 324mg e/c tablets	di1h.	04.07.01.00
	*aspirin 500mg m/r tablets	di19.	
	*aspirin 600mg e/c tablets	di1g.	04.07.01.00
	*aspirin 600mg tablets	di1i.	00.00.00.00
	*aspirin 75mg tablets	bu25.	02.09.00.00
	*caprin 300mg e/c tablets	di1k.	04.07.01.00
	*caprin 324mg e/c tablets	di1a.	04.07.01.00
	*caprin 75mg e/c tablets	bu2F.	02.09.00.00
	*disprin cv 100mg m/r tablets	bu28.	02.09.00.00
	*disprin cv 300mg m/r tablets	bu2a.	02.09.00.00
	aspirin 100mg effervescent tablets	bu21.	02.09.00.00
	aspirin 150mg suppositories	di1o.	04.07.01.00
	aspirin 162.5mg m/r capsules	bu2I.	02.09.00.00
	aspirin 300mg dispersible tablets	j112.	10.01.01.00
	aspirin 300mg e/c tablets	di1f.	04.07.01.00
	aspirin 300mg effervescent tablets	bu27.	02.09.00.00
	aspirin 300mg soluble tablets	di1m.	04.07.01.00
	aspirin 300mg suppositories	di1n.	04.07.01.00
	aspirin 300mg tablets	j111.	10.01.01.00
	aspirin 75mg dispersible tablets	di13.	
	aspirin 75mg dispersible tablets	bu23.	02.09.00.00
	aspirin 75mg e/c tablets	bu2B.	02.09.00.00
	aspirin 75mg soluble tablets	bu2c.	02.09.00.00
	aspirin [antiplatelet]	bu2..	02.09.00.00
	aspirin [central nervous system use]	di1..	04.07.01.00
	aspirin [cns] 300mg dispersible tablets	di12.	04.07.01.00
	aspirin [cns] 300mg tablets	di11.	04.07.01.00
	aspirin [musculoskeletal use]	j11..	10.01.01.00
	aspirin and the salicylates	j1...	10.01.01.00
	aspirin+metoclopramide 900mg/10mg/sachet powder	dl1b.	04.07.04.01
	aspirin+papaveretum 500mg/7.71mg dispersible tablets	diaG.	04.07.01.00
	aspirin/paracetamol/codeine tablets	dia1.	04.07.01.00
	dipyridamole+aspirin	bu4..	02.09.00.00
	dipyridamole+aspirin 200mg/25mg m/r capsules	bu41.	02.09.00.00
	disprin 300mg dispersible tablets	di1r.	04.07.01.00
	isosorbide mononitrate+aspirin	blm..	02.06.01.00
	isosorbide mononitrate+aspirin 60mg/150mg m/r tablets	blmy.	02.06.01.00
	isosorbide mononitrate+aspirin 60mg/75mg m/r tablets	blmz.	02.06.01.00
	nu-seals aspirin 300mg e/c tablets	di1c.	04.07.01.00
	nu-seals aspirin 600mg e/c tablets	di1d.	04.07.01.00
	nu-seals aspirin 75mg e/c tablets	bu2A.	02.09.00.00
	nu-seals cardio 75 e/c tablets	bu2G.	02.09.00.00

For peer review only - http://bmjopen.bmj.com/site/about/guidelines.xhtml

Supplementary Table 2: *CYP2C19* genotypes in UK Biobank

							UK Biobank						GnomAD	
**CYP2C19**	**Function**	**RSID**	**CHR**	**BP**	**A1**	**A2**	**HWE_p**	**INFO**	**MAF1**	**MAF1 %**	**note**		**MAF2**	**MAF2 %**
*2	LoF	rs4244285	10	96541616	G	A	0.9067	0.9998	0.1492	14.924	Imputed		0.1468	14.6800
*3	LoF	rs4986893	10	96540410	G	A	1.0000	1.0000	0.0001	0.006	Directly genotyped		0.0003	0.0264
*4	LoF	rs28399504	10	96522463	A	G	0.1615	0.7339	0.0022	0.216	Imputed		0.0025	0.2528
*5	LoF	rs56337013	10	96612495	C	T					Not in UKB imputed		0.0000	0.0008
*6	LoF	rs72552267	10	96535210	G	A					Not in UKB imputed		0.0003	0.0333
*7	LoF	rs72558186	10	96541756	T	C					Not in UKB imputed		0.0000	0.0000
*8	LoF	rs41291556	10	96535173	T	C	0.0415	1.0000	0.0030	0.304	Directly genotyped		0.0027	0.2710
*17	GoF	rs12248560	10	96521657	C	T	0.7279	0.9984	0.2154	21.539	Imputed		0.2314	23.1400

Function = LoF (Loss of Function, poor metaboliser), GoF (Gain of Function, rapid metaboliser)
BP = base position (hg19, build 37)
A1 = common allele
A2 = minor allele
HWE_p = Hardy-Weinberg deviation p-value
INFO = imputation quality score
MAF1 = minor allele frequency in UK Biobank Europeans
MAF2 = GnomAD European (non-Finnish) population minor allele frequency. GnomAD data can be viewed using URLs https://gnomad.broadinstitute.org/variant/rs4244285 and substituting the RSID for the appropriate variant.

For peer review only

Supplementary Table 3: *CYP2C19* associations with incident outcomes stratified by *17 genotype

Outcome / CYP2C19 genotype	N	N cases	Person-years	HR	95% CIs		p
Ischemic stroke							
Normal (*1/*1)	2,948	37	7,208				
Intermediate/poor (any *2-*8)	1,668	33	3,919	1.61	1.01	2.58	0.047
Poor or rapid heterozygote (*2–*8/*17)	476	8	1,191	1.25	0.58	2.69	0.570
Rapid (any *17)	2,385	32	6,039	0.99	0.62	1.59	0.970
Total	*7,477*	*110*	*18,358*				
Myocardial infarction							
Normal (*1/*1)	2,905	707	5,403				
Intermediate/poor (any *2-*8)	1,634	442	2,805	1.16	1.03	1.30	0.017
Poor or rapid heterozygote (*2–*8/*17)	466	112	904	0.98	0.81	1.20	0.880
Rapid (any *17)	2,354	562	4,691	0.95	0.85	1.06	0.330
Total	*7,359*	*1823*	*13,803*				

Analysis of European-ancestry participants with >1 clopidogrel prescription in the available GP prescribing data. Participants excluded if clopidogrel prescribing frequency was less than once every 2 months. Events <1 week after first clopidogrel prescription are excluded. Events occurring after the last known date of clopidogrel prescription are also excluded. HR=Hazard Ratios from Cox's proportional hazards regression models adjusted for age at first clopidogrel prescription, sex, and genetic principal components of ancestry 1-10. CI = Confidence Intervals.

For peer review only - http://bmjopen.bmj.com/site/about/guidelines.xhtml

STROBE 2007 (v4) Statement—Checklist of items that should be included in reports of *cohort studies*

Section/Topic	Item #	Recommendation	Reported on page #
**Title and abstract**	1	(*a*) Indicate the study's design with a commonly used term in the title or the abstract	1,2
		(*b*) Provide in the abstract an informative and balanced summary of what was done and what was found	2
**Introduction**			
Background/rationale	2	Explain the scientific background and rationale for the investigation being reported	4
Objectives	3	State specific objectives, including any prespecified hypotheses	4,5
**Methods**			
Study design	4	Present key elements of study design early in the paper	4-7
Setting	5	Describe the setting, locations, and relevant dates, including periods of recruitment, exposure, follow-up, and data collection	6
Participants	6	(*a*) Give the eligibility criteria, and the sources and methods of selection of participants. Describe methods of follow-up	6-8, Figure 1 (flowchart)
		(*b*) For matched studies, give matching criteria and number of exposed and unexposed	Na
Variables	7	Clearly define all outcomes, exposures, predictors, potential confounders, and effect modifiers. Give diagnostic criteria, if applicable	6-9
Data sources/ measurement	8*	For each variable of interest, give sources of data and details of methods of assessment (measurement). Describe comparability of assessment methods if there is more than one group	6-8
Bias	9	Describe any efforts to address potential sources of bias	8-9
Study size	10	Explain how the study size was arrived at	6-8
Quantitative variables	11	Explain how quantitative variables were handled in the analyses. If applicable, describe which groupings were chosen and why	8-9 (time to event, rather than quantitative)
Statistical methods	12	(*a*) Describe all statistical methods, including those used to control for confounding	8-9
		(*b*) Describe any methods used to examine subgroups and interactions	8-9
		(*c*) Explain how missing data were addressed	6-8, Figure 1 (flowchart)
		(*d*) If applicable, explain how loss to follow-up was addressed	Na

For peer review only

		(*e*) Describe any sensitivity analyses	8-9
**Results**			
Participants	13*	(a) Report numbers of individuals at each stage of study—eg numbers potentially eligible, examined for eligibility, confirmed eligible, included in the study, completing follow-up, and analysed	10, Figure 1 (cohort flowchart)
		(b) Give reasons for non-participation at each stage	10, Figure 1 (cohort flowchart)
		(c) Consider use of a flow diagram	Figure 1
Descriptive data	14*	(a) Give characteristics of study participants (eg demographic, clinical, social) and information on exposures and potential confounders	10. Table 1
		(b) Indicate number of participants with missing data for each variable of interest	10, Figure 1 (cohort flowchart)
		(c) Summarise follow-up time (eg, average and total amount)	10. Table 1
Outcome data	15*	Report numbers of outcome events or summary measures over time	10-12. Table 1 & 2
Main results	16	(*a*) Give unadjusted estimates and, if applicable, confounder-adjusted estimates and their precision (eg, 95% confidence interval). Make clear which confounders were adjusted for and why they were included	10-13. Absolute effects are unadjusted. Regression estimates adjusted for age and sex.
		(*b*) Report category boundaries when continuous variables were categorized	Na
		(*c*) If relevant, consider translating estimates of relative risk into absolute risk for a meaningful time period	10, 12
Other analyses	17	Report other analyses done—eg analyses of subgroups and interactions, and sensitivity analyses	10-12
**Discussion**			
Key results	18	Summarise key results with reference to study objectives	14
**Limitations**			
Interpretation	20	Give a cautious overall interpretation of results considering objectives, limitations, multiplicity of analyses, results from similar studies, and other relevant evidence	14-15
Generalisability	21	Discuss the generalisability (external validity) of the study results	15
**Other information**			
Funding	22	Give the source of funding and the role of the funders for the present study and, if applicable, for the original study on	16

For peer review only

		which the present article is based	

*Give information separately for cases and controls in case-control studies and, if applicable, for exposed and unexposed groups in cohort and cross-sectional studies.

Note: An Explanation and Elaboration article discusses each checklist item and gives methodological background and published examples of transparent reporting. The STROBE checklist is best used in conjunction with this article (freely available on the Web sites of PLoS Medicine at http://www.plosmedicine.org/, Annals of Internal Medicine at http://www.annals.org/, and Epidemiology at http://www.epidem.com/). Information on the STROBE Initiative is available at www.strobe-statement.org.

For peer review only

BMJ Open

Analysis of CYP2C19 genetic variants with ischaemic events in UK patients prescribed clopidogrel in primary care: a retrospective cohort study

Journal:	*BMJ Open*
Manuscript ID	bmjopen-2021-053905.R1
Article Type:	Original research
Date Submitted by the Author:	28-Sep-2021
Complete List of Authors:	Pilling, Luke; University of Exeter, Epidemiology and Public Health<br>Türkmen, Deniz; University of Exeter, Epidemiology and Public Health<br>Fullalove, Hannah; University of Exeter, Epidemiology and Public Health<br>Atkins, Janice; University of Exeter,<br>Delgado, Joao; University of Exeter, College of Medicine and Health<br>Kuo, Chia-Ling; UConn Health<br>Kuchel, George; University of Connecticut,<br>Ferrucci, Luigi; Harbor Hospital, Baltimore, National Institute on Aging NIA-ASTRA Unit<br>Bowden, Jack; University of Exeter, Epidemiology and Public Health<br>Masoli, Jane; University of Exeter, Epidemiology and Public Health<br>Melzer, David; University of Exeter, Epidemiology and Public Health
**Primary Subject Heading**:	Cardiovascular medicine
Secondary Subject Heading:	General practice / Family practice, Genetics and genomics, Epidemiology
Keywords:	Myocardial infarction < CARDIOLOGY, EPIDEMIOLOGY, GENETICS, Stroke < NEUROLOGY, PREVENTIVE MEDICINE

SCHOLARONE™
Manuscripts

BMJ

I, the Submitting Author has the right to grant and does grant on behalf of all authors of the Work (as defined in the below author licence), an exclusive licence and/or a non-exclusive licence for contributions from authors who are: i) UK Crown employees; ii) where BMJ has agreed a CC-BY licence shall apply, and/or iii) in accordance with the terms applicable for US Federal Government officers or employees acting as part of their official duties; on a worldwide, perpetual, irrevocable, royalty-free basis to BMJ Publishing Group Ltd ("BMJ") its licensees and where the relevant Journal is co-owned by BMJ to the co-owners of the Journal, to publish the Work in this journal and any other BMJ products and to exploit all rights, as set out in our licence.

The Submitting Author accepts and understands that any supply made under these terms is made by BMJ to the Submitting Author unless you are acting as an employee on behalf of your employer or a postgraduate student of an affiliated institution which is paying any applicable article publishing charge ("APC") for Open Access articles. Where the Submitting Author wishes to make the Work available on an Open Access basis (and intends to pay the relevant APC), the terms of reuse of such Open Access shall be governed by a Creative Commons licence – details of these licences and which Creative Commons licence will apply to this Work are set out in our licence referred to above.

Other than as permitted in any relevant BMJ Author's Self Archiving Policies, I confirm this Work has not been accepted for publication elsewhere, is not being considered for publication elsewhere and does not duplicate material already published. I confirm all authors consent to publication of this Work and authorise the granting of this licence.

For peer review only

Analysis of *CYP2C19* genetic variants with ischaemic events in UK patients prescribed clopidogrel in primary care: a retrospective cohort study

Luke C. Pilling[1,2], Deniz Türkmen[1], Hannah Fullalove[1], Janice L. Atkins[1], João Delgado[1], Chia-Ling Kuo[3], George A. Kuchel[2], Luigi Ferrucci[4], Jack Bowden[5], Jane A.H. Masoli[1,6], David Melzer[1,2]

1. Epidemiology and Public Health Group, College of Medicine and Health, University of Exeter, Exeter, UK

2. UConn Center on Aging, University of Connecticut, Farmington, CT, USA

3. Connecticut Convergence Institute for Translation in Regenerative Engineering, University of Connecticut, CT, USA

4. National Institute on Aging, Baltimore, MD, USA

5. Exeter Diabetes Group (ExCEED), College of Medicine and Health, University of Exeter, Exeter, UK

6. Department of Healthcare for Older People, Royal Devon and Exeter Hospital, Barrack Road, Exeter, UK.

Corresponding author

Dr Luke Pilling (L.Pilling@exeter.ac.uk)

Epidemiology and Public Health Group, University of Exeter, College House, St. Luke's Campus, Exeter, EX1 2LU, UK.

Word count: (Introduction/Methods/Results/Discussion) 4,523

For peer review only

Abstract

Objective To determine whether *CYP2C19* Loss of Function (LoF) alleles increase risk of ischaemic stroke and myocardial infarction (MI) in UK primary care patients prescribed clopidogrel.

Design Retrospective cohort analysis.

Setting Primary care practices in the UK from January 1999 to September 2017.

Participants 7,483 European-ancestry adults from the UK Biobank study with genetic and linked primary care data, aged 36 to 79 years at time of first clopidogrel prescription.

Interventions Clopidogrel prescription in primary care, mean duration 2.6 years (range 2 months to 18 years)

Main outcome measure Hospital inpatient-diagnosed ischaemic stroke, MI or angina whilst treated with clopidogrel.

Results 28.7% of participants carried at least one *CYP2C19* LoF variant. LoF carriers had higher rates of incident ischaemic stroke whilst treated with clopidogrel compared to those without the variants (8 per 1,000 person-years versus 5.2 per 1,000 person-years; Hazard Ratio 1.53: 95% Confidence Intervals 1.04 to 2.26, p=0.031). LoF carriers also had increased risk of MI (HR 1.14: 95%CI 1.04 to 1.26, p=0.008). In combined analysis LoF carriers had increased risk of any ischaemic event (stroke or MI) (HR 1.17: 95%CI 1.06 to 1.29, p=0.002). Adjustment for aspirin co-prescription produced similar estimates. In lifetables using observed incidence rates, 22.5% (95%CI 14.4% to 34.0%) of *CYP2C19* LoF carriers on clopidogrel were projected to develop an ischaemic stroke by age 79 (oldest age in the study), compared to 15.4% (95%CI 11.4% to 20.5%) in non-carriers, i.e. 7.1% excess stroke incidence in LoF carriers by age 79.

Conclusions A substantial proportion of the UK population carry genetic variants that reduce metabolism of clopidogrel to its active form. In family practice patients on clopidogrel, *CYP2C19* LoF variants are associated with substantially higher incidence of ischaemic events. Genotype-guided selection of antiplatelet medications may improve outcomes in patients carrying *CYP2C19* genetic variants.

For peer review only

For peer review only - http://bmjopen.bmj.com/site/about/guidelines.xhtml

Strengths and limitations of this study

- This study included a relatively large sample of 7,483 adults prescribed clopidogrel, and an extended follow-up period of up to 18 years
- The data are from a retrospective cohort in UK Biobank, which recruited a relatively healthy population at baseline
- Data were from linked electronic medical records (for prescribing and outcome data) and did not rely on potentially biased patient self-reporting
- Electronic record data on prescriptions in the community were analysed, but did not include hospital inpatient prescription data
- The sample had a maximum age of 79 years, so data on 80 plus year olds was not included

For peer review only - http://bmjopen.bmj.com/site/about/guidelines.xhtml

Introduction

Platelet aggregation, the process by which platelets adhere to each other, has long been recognized as critical for hemostatic plug formation and thrombosis (1). Antiplatelet therapy that reduces platelet aggregation has become a central part of treatment to reduce cardiovascular and cerebrovascular disease incidence in at-risk patient groups (2). Clopidogrel is an irreversible antagonist for the platelet $P2Y_{12}$ adenosine diphosphate receptor, thereby inhibiting platelet function and reducing likelihood of thrombosis (3). In 2018 it was the 39th most commonly prescribed drug in the USA (20 million prescriptions) (4). Clopidogrel is a prodrug that requires transformation to its bioactive form by cytochrome P-450 (CYP) enzymes, primarily CYP2C19 (3). Loss-of-function (LoF) genetic polymorphisms in *CYP2C19* impair clopidogrel metabolism, with carriers of any LoF variant reported to have a 32.4% reduction in plasma active metabolite levels compared to non-carriers (3). Clopidogrel resistance (high on-treatment platelet reactivity) is predominantly caused by *CYP2C19* LoF variants, with a recent study of clopidogrel on-treatment reactivity reporting that 71.7% of LoF carriers show clopidogrel resistance, compared to 32.1% of non-carriers (5). *CYP2C19* LoF carriers therefore have decreased platelet inhibition with consequently increased risk of thrombosis (6).

LoF variants in the *CYP2C19* gene are common in many populations, with the *2 allele ranging from 15% frequency in Europeans (with ~27% of the population carrying at least one copy) to >30% in Asian populations (7). A recent 2017 meta-analysis of 15 studies (12 East Asian, 3 European ancestry predominantly from the USA) included 4,762 stroke or transient ischaemic attack (TIA) patients treated with clopidogrel; patients carrying any *CYP2C19* LoF alleles had nearly double the rates of incident strokes (Risk Ratio [RR] 1.92: 95% Confidence Intervals 1.10 to 2.06, p=0.01) compared to non-carriers (8). However, most studies thus far focused on specific hospitalized patient groups with short (up to 1 year) follow-ups. A 2011 systematic review concluded that a significant association with stent thrombosis was due to small study effect bias and replication diversity (9). Although the US Food and Drug Administration added a Boxed Warning to the clopidogrel label regarding poor metabolizers due to *CYP2C19* variants (10) in 2010, there is continued debate about whether the magnitude of effect of these variants on clinical outcomes justifies *CYP2C19* genotype (6) guided prescription or use of alternative medications unaffected by these variants. In 2019 a consensus statement from experts in the field recommended genotyping as an optional tool for guiding treatment in certain scenarios (11).

For peer review only - http://bmjopen.bmj.com/site/about/guidelines.xhtml

Given the widespread continuing use of clopidogrel, and the current scarcity of evidence on clinical outcomes due to the LoF variants in the primary care setting, we aimed to estimate the association between *CYP2C19* LoF alleles and incident hospital-diagnosed ischaemic stroke and MI in UK Biobank participants on treatment with clopidogrel. We also aimed to estimate longer-term (>1 year) outcomes, as these have previously been understudied, and to estimate outcomes of non-*CYP2C19* variants reported to have similar effects (12).

For peer review only

For peer review only - http://bmjopen.bmj.com/site/about/guidelines.xhtml

Methods

UK Biobank cohort

The UK Biobank recruited 503,325 community-based volunteers aged 40-70 years who visited one of 22 assessment centers in England, Scotland or Wales between 2006 and 2010 (13). Data collected at the baseline assessment included extensive questionnaires on demographic, health, and lifestyle information. Anthropometric measures were also taken, in addition to blood samples for future biochemical and genetic analysis. The North West Multi-Centre Research Ethics Committee approved the collection and use of UK Biobank data (Research Ethics Committee reference 11/NW/0382). This research was conducted under UK Biobank application 14631 (PI: DM). UK Biobank volunteers tended to be healthier than the general population at baseline assessment (14), however this largely prospective analysis of linked primary care data is less affected by sample response patterns than baseline parameters.

Prescription data

General practice prescriptions data is currently available for 230,096 (45.7%) participants, with >57 million prescribing events recorded. UK Biobank obtained data from the three main primary care electronic clinical record data providers (England: TPP/Vision; Scotland and Wales: EMIS/Vision). The extract date differs between the supplier's computer systems, and we used the following censoring dates: June 2016 (England), April 2017 (Scotland), and September 2017 (Wales). Detailed description of the data extracted and limitations are available from UK Biobank (15). Data includes prescription dates, drug code (in clinical Read v2, British National Formulary (BNF), or Dictionary of Medicines and Devices format (depending on supplier), drug name, and quantity, where available. We identified all prescribing events for clopidogrel and aspirin. Details for drug names and clinical codes used are available in the Supplementary Information and Supplementary Table 1.

In the prescribing data we identified all records matching a clopidogrel code and for each patient determined the date first prescribed, date last prescribed, number of total prescriptions, and average number of prescriptions over the time period. We analyzed data for patients who received two or more prescriptions, with at least one clopidogrel prescription for each 2-month period between the dates first/last prescribed. This last criterion excluded participants with longer gaps between prescriptions, who may not have been taking clopidogrel for the entire study period, thus minimizing immortal time bias (16).

For peer review only - http://bmjopen.bmj.com/site/about/guidelines.xhtml

Participants never prescribed clopidogrel were used for sensitivity analysis of *CYP2C19* LoF variants and relevant vascular outcomes.

CYP2C19 genetic variants

Directly genotyped genetic variants (n=805,426) were available for 488,377 UK Biobank participants, based on two almost identical genotyping platforms sharing >95% of variants: the Affymetrix Axiom UKB array (in 438,427 participants) and the Affymetrix UKBiLEVE array (in 49,950 participants). After extensive quality control by the central UK Biobank team (17), genotype imputation was successful in 487,442 participants and increased the number of genetic variants to ~96 million (17). Our primary analysis included 451,367 participants (93%) identified as genetically European (identified by genetic clustering, as described previously (18)), to reduce bias in genetic studies from population stratification, which arises when different ancestral groups are analyzed together in genetic analyses (19). Secondary analysis in other ancestral groups was not possible due to low numbers in the clopidogrel patients (n=492 non-Europeans with sufficient clopidogrel data; this group included all other ancestries such as South or East Asian, African, and mixed).

We analyzed the *CYP2C19* genetic variants (*2-*8 and *17) with well-documented effects on clopidogrel metabolism in the literature (9) and in the PharmGKB database (12): three loss-of-function alleles (*2, *3 and *8; intermediate or poor metabolizers) and one gain-of-function allele (*17; rapid metabolizer) were either directly genotyped (*3 and *8) or imputed with high confidence (*2 and *17: >99.9% imputation confidence). *CYP2C19**4 was imputed with <80% imputation confidence (74.4%) so was excluded from analysis (*4 is very rare so this has minimal impact on the analysis: with 0.25% allele frequency reported in the GnomAD (7) database https://gnomad.broadinstitute.org/). *CYP2C19* *5, *6 and *7 were not available in the genotyping data, as they are extremely rare, especially in European populations (<0.1% allele frequency in GnomAD (7)). See Supplementary Table 2 for details on individual variants used.

Non-*CYP2C19* genetic variants

We also investigated non-*CYP2C19* genetic variants that affect clopidogrel from two sources: we searched the PharmGKB database (12) for variants classified as being supported by moderate or high clinical annotation levels of evidence, i.e. associations that replicated in subsequent studies, even if the original study was small. In addition to variants in *CYP2C19* (level 1 "high" levels of evidence) one variant in *CES1* has level 2B "moderate" evidence:

For peer review only - http://bmjopen.bmj.com/site/about/guidelines.xhtml

rs71647871 was directly genotyped on the microarray but is rare, with only 249 heterozygotes in the clopidogrel analysis group (no homozygotes).

Lastly, we investigated variants identified in a genome-wide association study of clopidogrel active metabolite levels (rs187941554 and rs80343429) (20). Both variants are available in the UK Biobank imputed genotype data: rs187941554 was imputed with high confidence (99.9%), however the overall rs80343429 imputation accuracy was 81.7% (n=3 individual participants with low-confidence genotype calls - imputed genotype dose >0.25 and <0.75 - were excluded from analysis).

Disease ascertainment

Diagnosis of ischaemic stroke and MI ascertainment from hospital admissions records (Hospital Episode Statistics, HES) were available up to 14 years follow-up after baseline assessment, covering the entire period up to the date of censoring of primary care prescribing data (HES in England up to 30 September 2020: data from Scotland and Wales censored to 31 August 2020 and 28 Feb 2018, respectively). Diagnosis of ischaemic stroke was ascertained using ICD-10 code I63*. Diagnosis of MI was ascertained using ICD-10 codes I21*; I22*; I23*; I24*; I25*. In sensitivity analysis we investigated major bleeding using ICD-10 codes from (21) (see Supplementary Table 3 for codes used).

For prevalent ascertainment of diagnoses at baseline of transient ischaemic attack (TIA), MI/angina, atrial fibrillation, hypertension or peripheral vascular disease we used primary care records. Diagnosis codes (read v2) were downloaded from the CALIBER phenotype library (https://portal.caliberresearch.org/disease-or-syndrome) (22). Corresponding CTV3 codes were identified using the UK Biobank “Clinical coding classification systems and maps” resource (https://biobank.ctsu.ox.ac.uk/crystal/refer.cgi?id=592).

Statistical analysis

Data are from multiple sources (demographic and genetic data from UK Biobank assessment, clopidogrel prescribing data from linked electronic primary care records, and stroke and MI outcomes from linked electronic hospital inpatient records). Patients with any missing data needed for analysis were excluded, with details shown in the results.

Estimates of associations between genotype and incident disease were obtained from Cox’s proportions hazards regression models, with adjustment for age at first prescription, sex, and the first ten principal components of genetic ancestry (to control for population substructure).

For peer review only - http://bmjopen.bmj.com/site/about/guidelines.xhtml

Patients entered the model 7 days after the date of first prescription of clopidogrel; this was to exclude the small number of events that occurred immediately after being prescribed clopidogrel, before it could reasonably have had a preventative effect. Seven days was chosen because the effect of clopidogrel on platelet aggregation was significantly different between *CYP2C19* genotype groups after 7-10 days in a study of 375 patients (23). In sensitivity analysis we included all events during the prescribing period. Patients exited the model on the last known date of clopidogrel prescription. STATA (v15.1) software was used for analysis. We visually inspected Kaplan-Meier plots and used STATA function `estat phtest, detail` to generate Schoenfeld residuals to test for violations of the proportional-hazards assumption.

Population attributable fraction in this analysis is the estimated proportion of all cases that would not have occurred if no participants carried the clopidogrel modifying genetic variants. It was calculated by multiplying the proportion of incident ischaemic strokes in the genotype carriers by (1 – 1 / Hazard Ratio for ischaemic stroke) and similarly for MI (24). This method is preferred to simple observed vs. expected cases as it uses the estimate from the time-to-event model, which has been adjusted for covariates (24). Lifetable probabilities of incident outcomes were estimated from age 37 to 79 (the minimum and maximum ages the patients were first prescribed clopidogrel, respectively) by *CYP2C19* genotype, applying observed incidence rates in each age group to a notional cohort (where all participants in the analysis had reached the maximum age group), estimating cumulative incident case numbers, using STATA command `sts list`.

Patient and public involvement

Patients and participants were and are extensively involved in the UK Biobank study itself. No patients were involved in developing the research question or the outcomes tested. A lay abstract was reviewed by members of the Peninsula Public Engagement Group ("PenPEG") in Exeter, who provided positive feedback on the importance of the study from a patient perspective.

For peer review only - http://bmjopen.bmj.com/site/about/guidelines.xhtml

Results

7,483 UK Biobank participants of European ancestry met inclusion criteria, with at least two prescriptions of clopidogrel recorded in primary care data (see Figure 1 for cohort flowchart with detailed inclusion/exclusion criteria). The mean age at first prescription was 64.3 years (SD 7.2), and 2,563 were female (34.2%). The clopidogrel exposure period ranged from 2 months to 18 years (mean 2.59 years, SD 2.98). Cohort selection is described in Table 1. Patients carrying at least one *CYP2C19* LoF variant (*2-*8, intermediate/poor clopidogrel metabolizers) did not have significantly different likelihood of receiving clopidogrel compared to non-carriers (Odds Ratio 1.01: 95% Confidence Intervals 0.96 to 1.05, p=0.75) and had similar prevalence of cardiovascular co-morbidities prior to diagnosis (Table 1).

CYP2C19 LoF associations with incident ischaemic stroke

Incident hospital-diagnosed ischaemic stroke (cerebral infarction) was identified in 110 of 7,477 participants (1.47%) eligible for analysis (after excluding n=6 who had stroke within 7 days of first prescription). *CYP2C19* LoF (*2-*8, intermediate/poor metabolizers) carriers (n=2,144, 28.7%) were more likely to have an ischaemic stroke whilst on treatment for clopidogrel than patients without *CYP2C19* LoF genetic variants (Hazard Ratio (HR) 1.53: 95% CI 1.04 to 2.26, p=0.031). See Table 2 for details and Figure 2A for Kaplan-Meier plot. 1.29% of the patients without any *CYP2C19* LoF genetic variants had an stroke during the prescribing period (mean 2.59 years between first and last clopidogrel prescription, SD 2.98) compared to 1.91% of the *CYP2C19* LoF carriers: this absolute 0.64% excess is statistically significant (95% CI 0.021% to 1.35%, p=0.027).

To test whether the effect of *CYP2C19* LoF genotypes on ischaemic stroke risk was specific to clopidogrel, and not via a separate but unknown biological mechanism, we analyzed the 198,868 UK Biobank participants with GP prescribing data in whom clopidogrel was never prescribed (see Figure 1). There were 512 ischaemic stroke events in this group after baseline assessment and before the end of Feb 2016 (earliest censoring date for GP prescribing data). *CYP2C19* LoF carriers were not at significantly increased risk of ischaemic strokes compared to non-carriers (HR 1.12: 95% CI 0.93 to 1.35, p=0.24).

To calculate the population attributable fraction (PAF) we multiplied the proportion of incident ischaemic strokes that occurred in that patients who had intermediate or low metabolizer variants (41/110) by (1-1/HR for ischaemic stroke = 1-1/1.53 = 0.346) giving 0.129 (see Methods). We therefore estimate the PAF for ischaemic stroke - the proportion of ischaemic strokes in this population that would not have occurred if clopidogrel efficacy was not affected

For peer review only - http://bmjopen.bmj.com/site/about/guidelines.xhtml

by *CYP2C19* LoF variants - to be 12.9% (95% CI 1.4% to 20.8%). Kaplan-Meier curves for diagnosis of ischaemic strokes in participants prescribed clopidogrel are shown in Figure 2. In lifetable estimates based on observed incidence rates from 36 to 79 (the minimum and maximum ages at which clopidogrel was first prescribed) the risk of ischaemic stroke in CYP2C19 LoF carriers was 22.5% (95% CI 14.4% to 34.0%), compared with 15.4% (95% CI 11.4% to 20.5%) in non-carriers.

We performed secondary analyses estimating outcomes for participants prescribed clopidogrel for >1 year (n=4,316: 57.8%). *CYP2C19* LoF carriers had significantly increased risk of strokes (HR 1.63: 95% CI 1.01 to 2.64, p=0.047) in this period (Table 2). However the association with only those events within the first year of prescription was not statistically significant (HR 1.33: 95% CI 0.69 to 2.57, p=0.390). In tests of the proportional-hazards assumption (see Methods) in the model using all available events there was no evidence that the hazard ratio changed over time (p=0.42). The analysis using all participants suggests LoF carriers are at increased risk for the duration of the clopidogrel prescribing (HR 1.53: 95% CI 1.04 to 2.26, p=0.031).

In sensitivity analysis we adjusted the main analysis of *CYP2C19* LoF carriers for history of stroke, TIA, MI/angina, PAD, and atrial fibrillation to assess whether disease indication modified the effect of genotype on ischaemic stroke risk and found the estimate to be unaffected (HR 1.56: 95% CI 1.06 to 2.31, p=0.024). We also performed analysis including all events during the prescribing period (the primary analysis excluded events in the first 7 days of prescribing because the effect of clopidogrel on platelet aggregation was significantly different between *CYP2C19* genotype groups after 7-10 days in a study of 375 patients (23)). This period included 6 strokes and changed the estimates from (HR 1.53: 95% CI 1.04 to 2.26, p=0.031) to (HR 1.45: 95% CI 0.99 to 2.13, p=0.053). Though the 95% CIs cross the null the association is highly consistent, despite inclusion of some possibly invalid events.

We investigated hemorrhagic strokes (ICD-10 codes I61* and I62*), however there were too few diagnoses made (n=9 in the 7,483 participants included in the study) during the clopidogrel prescribing period, so no analysis was performed.

We also analyzed outcomes for the *17 (gain of function) genotype carriers (rapid metabolizers). *17 carriers were not at altered risk of ischaemic stroke during the prescribing period (HR 0.98: 95% CI 0.61 to 1.57, p=0.930, Supplementary Table 4) compared to *1/*1 patients. In analysis of Major Bleeding whilst prescribed clopidogrel *CYP2C19* *17 carriers were not at significantly greater or lesser risk compared to non-carriers (n events during prescribing period=167; OR 0.88: 95% CI 0.62 to 1.24, p=0.46).

For peer review only - http://bmjopen.bmj.com/site/about/guidelines.xhtml

CYP2C19 LoF associations with incident myocardial infarction

Incident hospital-diagnosed myocardial infarctions (MI) occurred in 1,823 of 7,359 participants (24.8%), after excluding 124 subjects who had MIs within 7 days of first clopidogrel prescription. *CYP2C19* LoF (*2-*8, intermediate/poor metabolizers) carriers (n=2,100, 28.5%) were more likely to have an MI whilst being prescribed clopidogrel than patients without *CYP2C19* LoF genetic variants (HR 1.14: 95% CI 1.04 to 1.26, p=0.008, see Table 2 for details and Figure 2B for Kaplan-Meier plot). The PAF for MI was 3.73% (95% CI 1.2% to 6.3%).

We performed secondary analysis investigating the participants prescribed clopidogrel for >1 year (n=3,511: 47.7%). *CYP2C19* LoF carriers were not at significantly increased risk of MIs (HR 1.09: 95% CI 0.90 to 1.33, p=0.37) in this period (Table 2). However, LoF carriers did have significantly increased risk of MI within the first year of clopidogrel prescription (HR 1.16: 95% CI 1.03 to 1.31, p=0.011). In tests of the proportional-hazards assumption (see Methods) in the model using all available events there was no evidence that the hazard ratio changed over time (p=0.70). The analysis using all participants suggests LoF carriers are at increased risk for the duration of the clopidogrel prescribing (HR 1.14: 95% CI 1.04 to 1.26, p=0.008).

We also analyzed the *17 (gain of function) genotype carriers (rapid metabolizers). *17 carriers did not have significantly different risk of MI during the prescribing period (HR 0.95: 95% CI 0.85 to 1.06, p=0.33, Supplementary Table 4) compared to *1/*1 patients.

The association between *CYP2C19* LoF carriers and MI in participants never prescribed clopidogrel (n=4,390 MIs) was not significant (HR 1.03: 95% CI 0.96 to 1.10, p=0.41). Sensitivity analysis including all MIs during the prescribing period (i.e. not excluding those within 7 days of first known prescription) included 126 more events and changed the estimates from (HR 1.14: 95% CI 1.04 to 1.26, p=0.008) to (HR 1.16: 95% CI 1.06 to 1.28, p=0.021).

In secondary analysis of any ischaemic event (stroke or MI, *n*=1,886 events in 7,354 patients included in analysis) *CYP2C19* LoF (*2-*8, intermediate/poor metabolizers) carriers were more likely to have an event whilst being prescribed clopidogrel than patients without *CYP2C19* LoF genetic variants (HR 1.17: 95% Cis 1.06 to 1.29, p=0.002).

Dual antiplatelet therapy: clopidogrel co-prescribing with aspirin

Of 7,483 patients prescribed clopidogrel 3,730 (49.9%) were also prescribed aspirin at least once during the analysis period. *CYP2C19* LoF carriers had slightly higher likelihood of ever receiving a prescription of aspirin compared to non-carriers during their clopidogrel exposure period (n=1,118 (52.2%) vs. n=2,629 (49.3%), odds ratio (OR) from logistic regression 1.14:

For peer review only - http://bmjopen.bmj.com/site/about/guidelines.xhtml

95% CI 1.02 to 1.26, p=0.017). Ever being prescribed aspirin did not significantly interact with *CYP2C19* LoF carrier status in the model of incident stroke or MI (p>0.05). The association between *CYP2C19* LoF carrier status and stroke or MI was consistent (albeit attenuated due reduced sample sizes in subgroups) in participants who received an aspirin prescription (n=3,730. Stroke HR 1.61: 95% CI 0.87 to 2.98, p=0.13; MI HR 1.10; 95% CI 0.98 to 1.24, p=0.10) and those who did not (n=3,753. Stroke HR 1.48: 95% CI 0.89 to 2.46, p=0.13; MI HR 1.19: 95% CI 0.98 to 1.45, p=0.087).

Analysis of non-*CYP2C19* genetic variation associated with clopidogrel active metabolite levels

A rare variant in *CES1* (rs71647871, n=249 CT heterozygotes) with moderate clinical annotation evidence for clopidogrel on the PharmGKB database (12) was not significantly associated with strokes or MIs (stroke HR 0.86: 95% CI 0.27 to 2.70, p=0.79; MI HR 1.26: 95% CI 0.99 to 1.60, p=0.057), although the MI association was borderline.

Two genetic variants identified in a GWAS of clopidogrel active metabolite levels (20) were also analyzed. Despite its low frequency in the population, rs187941554 (n=32 GA heterozygotes, 0.4% of 7,468) was associated with substantially raised risk of incident stroke (n strokes=2; HR 5.44: 95% CI 1.33 to 22.30, p=0.019) but not MIs (HR 1.20: 95% CI 0.62 to 2.32, p=0.58). The rs80343429 A allele was not associated with increased risk of stroke or MI (stroke HR 1.19: 95% CI 0.68 to 2.09, p=0.53; MI HR 1.09: 95% CI 0.94 to 1.26, p=0.25).

For peer review only

For peer review only - http://bmjopen.bmj.com/site/about/guidelines.xhtml

Discussion

Clopidogrel requires metabolic activation to be effective, mainly by the CYP2C19 liver enzyme. *CYP2C19* LoF genetic variants (implying intermediate or low metabolizer status) have been shown to increase stroke and MI rates mainly in hospitalized patients with acute ischaemic conditions (or undergoing related interventions such as cardiac stents (25)), with little data on outcomes in primary care or over longer follow-up periods. In this study of 7,483 UK Biobank primary care patients prescribed clopidogrel, carriers of *CYP2C19* LoF alleles had substantially increased risks of incident ischaemic strokes and myocardial infarction. We also showed that carriers of LoF alleles are at increased risk of stroke even during longer-term (>1 year) therapy; the effect is not limited to acute stroke patients.

Our results are consistent with previous studies of clopidogrel efficacy in *CYP2C19* LoF genotype carriers, although the previous evidence was predominantly from high-risk acutely hospitalized patient groups (26–29), with limited follow-up periods (rarely over 12 months). Some studies reported that the effect of *CYP2C19* genotype on reducing clopidogrel efficacy was limited to the short-term (1-6 months) only (30,31), but in our community based longer term follow-up we found no evidence for deviation from the proportional hazards assumptions, with survival analysis results suggesting sustained effects over the follow-up period. We had limited sample size and therefore limited statistical power to estimate effects for specific periods, but the intermediate or low metabolizer variant association with excess strokes was significant in the subgroup prescribed clopidogrel for more than one year.

A recent 2019 meta-analysis of 16 randomized clinical trials (32) found that dual antiplatelet therapy with aspirin was associated with significantly lower stroke rates (RR 0.80: 0.72 to 0.89), however some evidence of increased major bleeding was also observed (RR 1.90: 1.33 to 2.72). The authors noted that common *CYP2C19* LoF variants may account for the effectiveness of dual therapy over clopidogrel monotherapy (32). In our study we found LoF carriers still had increased stroke or MI risk, even if co-prescribed aspirin.

Clopidogrel is a recommended antiplatelet medication for the prevention of stroke in the USA (2) and is the currently recommended antiplatelet medication for prevention of stroke or TIA in the UK (33). However, antiplatelet medications such as ticagrelor and prasugrel are not dependent on CYP2C19 metabolism and reportedly have better clinical outcomes in LoF carriers compared to clopidogrel (34). A recent meta-analysis of 16 studies (8 RCTs and 8 cohorts) concluded that genotype-guided antiplatelet treatment (primarily based on *CYP2C19* LoF alleles) improved patient outcomes for major cardiovascular events (e.g. Relative Risk for MI 0.45: 95% CI 0.35 to 0.58, p<0.00001), with decreased risk of major bleeding (29). Treatment strategy in the different metabolizer groups varied between individual studies, but

For peer review only - http://bmjopen.bmj.com/site/about/guidelines.xhtml

included use of higher doses of clopidogrel (150 mg/d instead of the usual 75 mg/d), or prescribing of prasugrel or ticagrelor, or a combination. Other recent studies in different populations, especially in patients with acute coronary syndrome and those undergoing primary percutaneous coronary intervention such as the large POPular Genetics trial, also support this conclusion (26–28,35,36). Therefore, further work is needed in primary care populations to inform optimal antiplatelet care, given our estimate that over 12% of strokes in the UK study population on clopidogrel could be avoided if low and intermediate metabolizers could achieve the same clinical outcomes as are being achieved in the normal metabolizer group. Economic analysis in the UK comparing clopidogrel (out of patent at the time of writing) to alternatives not necessarily out of patent are needed.

This is the largest single study of primary care patients prescribed clopidogrel, incorporating *CYP2C29* genetic data and longer (>1 year) follow-up of clinical outcomes. However, this study is not without limitations. UK Biobank participants tended to be healthier than the general population at baseline assessment (14), however, linked primary care data used follow-up analysis should be unaffected by sample response patterns. Population attributable risk may be underestimated, as UK Biobank has a limited age range (mean age of 64 years at first prescription, max 79) that does not include many older people at high risk of strokes or MIs, so estimates of absolute risk may be greater in the general population. Further work is required to model this in other countries. A further limitation is that data from patients treated soon after a stroke are limited by the absence of information on hospital prescribing and the inclusion of patients who received 2 or more consecutive clopidogrel prescriptions in the community, consistent with our focus on longer term outcomes in the community. The prescribing data from primary care is designed to be comprehensive for community-based subjects. The only other route to receipt of clopidogrel is for subjects who had periods as hospital inpatients, but in the UK system care is routinely handed back to primary care on hospital discharge, with very short term (usually up to 7 days) medication prescribed. The availability of only primary care prescribing (with no hospital prescribing details available) may mean that some clopidogrel prescriptions have been missed and therefore some patients or exposure periods excluded, but likely only for periods during and soon after hospital admissions. In our primary analysis we excluded events within 7 days of first known prescription, to give clopidogrel time to have a preventative effect: in sensitivity analysis we included these events as patients may have received a prescription before their first in primary care, and we found that results were highly consistent, albeit with modestly altered estimates. Although this is a relatively large study of 7,483 patients, stratified analyses (such as *2 heterozygous/homozygous groups, or by aspirin co-prescribing) were underpowered. The analysis was restricted to UK Biobank participants of genetically European ancestry (93% of the cohort); only 492 non-European

For peer review only - http://bmjopen.bmj.com/site/about/guidelines.xhtml

participants had sufficient clopidogrel data, but this included all other ancestries including South or East Asian, African, and mixed, so analysis was not possible. Future analyses with more data will be needed to address these research questions. Similarly, analyses of the low frequency variants in larger samples may be needed to refine estimates, including for rs187941554, for which our nominally significant association (HR 5.44: 95% CIs 1.33 to 22.30, p=0.019) was based on only two strokes in 32 subjects.

To conclude, intermediate and low metabolizers (due to *CYP2C19* loss of function alleles) who were prescribed clopidogrel in the primary care setting had substantially increased risks of incident hospital-diagnosed strokes and MIs, compared to normal metabolizers. Evidence for improved patient outcomes with *CYP2C19* genotype-guided antiplatelet therapy is increasing, especially in the acute setting. Genotype guided section of antiplatelet medications may improve outcomes for primary care patients carrying *CYP2C19* variants.

For peer review only

For peer review only - http://bmjopen.bmj.com/site/about/guidelines.xhtml

Acknowledgements

Access to UK Biobank resource was granted under Application Number 14631. We would like to thank UK Biobank participants and coordinators for this dataset. The authors would like to acknowledge the use of the University of Exeter High-Performance Computing (HPC) facility in carrying out this work.

Author Contributions

LCP performed the analysis, interpreted results, created the figures, searched literature, and co-wrote the manuscript. DT and HF performed the literature search, performed analyses, and contributed to the manuscript. JA and JD performed analyses and contributed to the manuscript. CK, GAK, LF and JB contributed to data interpretation and contributed to the manuscript. JM provided expert clinical interpretation of the data and contributed to the manuscript. DM oversaw data analysis, interpretation, and literature searching, and led the writing of the manuscript. DM is the guarantor. The corresponding author attests that all listed authors meet authorship criteria and that no others meeting the criteria have been omitted.

Funding

LCP and DM are supported by the University of Exeter Medical School and the University of Connecticut School of Medicine. DT is funded by the Ministry of National Education, Republic of Turkey. JA was supported by an award to DM by the UK Medical Research Council (MR/S009892/1). JD is also supported by the Alzheimer's Society [grant: 338 (AS-JF-16b-007)]. GAK is supported by the Travelers Chair in Geriatrics and Gerontology. JB is funded by an Expanding Excellence in England (E3) research grant awarded to the University of Exeter. LF is supported by the National Institute on Aging, NIH. JM is funded by a National Institute for Health Research Fellowship(NIHR301445). This publication presents independent research funded by the National Institute for Health Research (NIHR). The views expressed are those of the author(s) and not necessarily those of the NHS, the NIHR or the Department of Health and Social Care. The funders had no input in the study design; in the collection, analysis, and interpretation of data; in the writing of the report; or in the decision to submit the article for publication. The researchers acted independently from the study sponsors in all aspects of this study.

Competing interests

All authors have completed the ICMJE uniform disclosure form (at www.icmje.org/coi_disclosure.pdf) and declare: no support from any organisation for the submitted work; no financial relationships with any organisations that might have an interest

For peer review only

For peer review only - http://bmjopen.bmj.com/site/about/guidelines.xhtml

in the submitted work in the previous three years; no other relationships or activities that could appear to have influenced the submitted work.

Ethical approval

The North West Multi-Centre Research Ethics Committee approved the collection and use of UK Biobank data (Research Ethics Committee reference 11/NW/0382).

Data sharing

Data are available on application to the UK Biobank (www.ukbiobank.ac.uk/register-apply). Additional data are available from the corresponding author on reasonable request.

Transparency

The lead author (DM) affirms that this manuscript is an honest, accurate, and transparent account of the study being reported; that no important aspects of the study have been omitted; and that any discrepancies from the study as planned (and, if relevant, registered) have been explained.

Supplementary Material

Supplementary Methods

Supplementary Tables 1-4

For peer review only

References

1. Jackson S. The growing complexity of platelet aggregation. Blood [Internet]. 2007;109(12):5087–95.

2. Powers WJ, Rabinstein AA, Ackerson T, Adeoye OM, Bambakidis NC, Becker K, et al. 2018 Guidelines for the Early Management of Patients With Acute Ischemic Stroke: A Guideline for Healthcare Professionals From the American Heart Association/American Stroke Association. Stroke. 2018;49(3):e46–110.

3. Mega JL, Close SL, Wiviott SD, Shen L, Hockett RD, Brandt JT, et al. Cytochrome P-450 Polymorphisms and Response to Clopidogrel. N Engl J Med [Internet]. 2009 Jan 22 [cited 2020 Jul 8];360(4):354–62.

4. Kane SP. ClinCalc DrugStats: Clopidogrel Bisulphate [Internet]. 2021 [cited 2021 Jan 20].

5. Fu H, Hu P, Ma C, Peng F, He Z. Association of clopidogrel high on-treatment reactivity with clinical outcomes and gene polymorphism in acute ischemic stroke patients. Medicine (Baltimore) [Internet]. 2020 Apr;99(15):e19472.

6. Simon T, Danchin N. Clinical Impact of Pharmacogenomics of Clopidogrel in Stroke. Circulation [Internet]. 2017 Jan 3;135(1):34–7.

7. Karczewski KJ, Francioli LC, Tiao G, Cummings BB, Alföldi J, Wang Q, et al. The mutational constraint spectrum quantified from variation in 141,456 humans. Nature. 2020;581(7809):434–43.

8. Pan Y, Chen W, Xu Y, Yi X, Han Y, Yang Q, et al. Genetic Polymorphisms and Clopidogrel Efficacy for Acute Ischemic Stroke or Transient Ischemic Attack. Circulation [Internet]. 2017 Jan 3;135(1):21–33.

9. Bauer T, Bouman HJ, Van Werkum JW, Ford NF, Ten Berg JM, Taubert D. Impact of CYP2C19 variant genotypes on clinical efficacy of antiplatelet treatment with clopidogrel: Systematic review and meta-analysis. BMJ. 2011;343(7819):1–18.

10. U.S. Food & Drug Administration. FDA Drug Safety Communication: Reduced effectiveness of Plavix (clopidogrel) in patients who are poor metabolizers of the drug [Internet]. 2010 [cited 2021 Mar 29].

11. Sibbing D, Aradi D, Alexopoulos D, ten Berg J, Bhatt DL, Bonello L, et al. Updated Expert Consensus Statement on Platelet Function and Genetic Testing for Guiding P2Y12 Receptor Inhibitor Treatment in Percutaneous Coronary Intervention. JACC

For peer review only - http://bmjopen.bmj.com/site/about/guidelines.xhtml

Cardiovasc Interv [Internet]. 2019 Aug;12(16):1521–37.

12. Whirl-Carrillo M, McDonagh EM, Hebert JM, Gong L, Sangkuhl K, Thorn CF, et al. Pharmacogenomics Knowledge for Personalized Medicine. Clin Pharmacol Ther [Internet]. 2012 Oct;92(4):414–7.

13. Sudlow C, Gallacher J, Allen N, Beral V, Burton P, Danesh J, et al. UK Biobank: An Open Access Resource for Identifying the Causes of a Wide Range of Complex Diseases of Middle and Old Age. PLoS Med. 2015;12(3):e1001779.

14. Fry A, Littlejohns TJ, Sudlow C, Doherty N, Adamska L, Sprosen T, et al. Comparison of Sociodemographic and Health-Related Characteristics of UK Biobank Participants With Those of the General Population. Am J Epidemiol. 2017 Nov 1;186(9):1026–34.

15. UK Biobank. UK Biobank Primary Care Linked Data Version 1.0 [Internet]. 2019 [cited 2019 Sep 30].

16. Yadav K, Lewis RJ. Immortal Time Bias in Observational Studies. JAMA [Internet]. 2021 Feb 16;325(7):686.

17. Bycroft C, Freeman C, Petkova D, Band G, Elliott LT, Sharp K, et al. The UK Biobank resource with deep phenotyping and genomic data. Nature. 2018;562(7726):203–9.

18. Thompson WD, Tyrrell J, Borges M-C, Beaumont RN, Knight BA, Wood AR, et al. Association of maternal circulating 25(OH)D and calcium with birth weight: A mendelian randomisation analysis. PLoS Med [Internet]. 2019 Jun;16(6):e1002828.

19. Hellwege JN, Keaton JM, Giri A, Gao X, Velez Edwards DR, Edwards TL. Population Stratification in Genetic Association Studies. Curr Protoc Hum Genet [Internet]. 2017 Oct 13;95(1).

20. Backman JD, O'Connell JR, Tanner K, Peer CJ, Figg WD, Spencer SD, et al. Genome-wide analysis of clopidogrel active metabolite levels identifies novel variants that influence antiplatelet response. Pharmacogenet Genomics [Internet]. 2017 Apr [cited 2020 Jul 1];27(4):159–63.

21. Bakhai A, Petri H, Vahidnia F, Wolf C, Ding Y, Foskett N, et al. Real-world data on the incidence, mortality, and cost of ischaemic stroke and major bleeding events among non-valvular atrial fibrillation patients in England. J Eval Clin Pract [Internet]. 2021 Feb 21;27(1):119–33.

22. Kuan V, Denaxas S, Gonzalez-Izquierdo A, Direk K, Bhatti O, Husain S, et al. A chronological map of 308 physical and mental health conditions from 4 million

For peer review only

For peer review only - http://bmjopen.bmj.com/site/about/guidelines.xhtml

individuals in the English National Health Service. Lancet Digit Heal [Internet]. 2019 Jun;1(2):e63–77.

23. Yi X, Lin J, Wang Y, Zhou Q, Wang C, Cheng W, et al. Association of cytochrome P450 genetic variants with clopidogrel resistance and outcomes in acute ischemic stroke. J Atheroscler Thromb. 2016;23(10):1188–200.

24. Mansournia MA, Altman DG. Population attributable fraction. BMJ [Internet]. 2018 Feb 22;k757.

25. Pereira NL, Rihal C, Lennon R, Marcus G, Shrivastava S, Bell MR, et al. Effect of CYP2C19 Genotype on Ischemic Outcomes During Oral P2Y12 Inhibitor Therapy. JACC Cardiovasc Interv [Internet]. 2021 Mar;1–12.

26. Ramesh S, Socrates S, Rajasekaran MA, Senguttuvan N. Incidence of major adverse cardiovascular events with genotype test guided antiplatelet treatment strategy after percutaneous coronary intervention. Indian Heart J [Internet]. 2020 Nov;72(6):589–92.

27. Zhang M, Wang J, Zhang Y, Zhang P, Chao Y, Gao M, et al. Effects of individualized antiplatelet therapy, based on CYP2C19 genotyping, on platelet function in patients underwent percutaneous coronary intervention. Perfusion [Internet]. 2020 Dec 10;026765912097858.

28. Russmann S, Rahmany A, Niedrig D, Hatz K-D, Ludin K, Burden AM, et al. Implementation and management outcomes of pharmacogenetic CYP2C19 testing for clopidogrel therapy in clinical practice. Eur J Clin Pharmacol [Internet]. 2020 Nov 26;

29. Zhang H, Xiang Q, Liu Z, Mu G, Xie Q, Zhou S, et al. Genotype-guided antiplatelet treatment versus conventional therapy: A systematic review and meta-analysis . Br J Clin Pharmacol. 2020;(October):1–17.

30. Wang Y, Cai H, Zhou G, Zhang Z, Liu X. Effect of CYP2C19*2 and *3 on clinical outcome in ischemic stroke patients treated with clopidogrel. J Neurol Sci [Internet]. 2016 Oct;369:216–9.

31. G. C, G. P, P. F, B. L, M. T, M. M, et al. Prospective evaluation of on-clopidogrel platelet reactivity over time in patients treated with percutaneous coronary intervention: Relationship with gene polymorphisms and clinical outcome. J Am Coll Cardiol [Internet]. 2011;57(25):2474–83.

32. Kheiri B, Osman M, Abdalla A, Haykal T, Swaid B, Ahmed S, et al. Clopidogrel and aspirin after ischemic stroke or transient ischemic attack: an updated systematic

For peer review only

For peer review only - http://bmjopen.bmj.com/site/about/guidelines.xhtml

review and meta-analysis of randomized clinical trials. J Thromb Thrombolysis [Internet]. 2019;47(2):233–47.

33. NICE. NICE (UK National Institute for Health and Care Excellence) guidelines - antiplatelet treatment (September 2018 guidance) [Internet]. 2018 [cited 2020 Jun 26].

34. Yoon HY, Lee N, Seong J, Gwak HS. Efficacy and safety of clopidogrel versus prasugrel and ticagrelor for coronary artery disease treatment in patients with CYP2C19 LoF alleles: a systemic review and meta-analysis. Br J Clin Pharmacol [Internet]. 2020 Aug 28;86(8):1489–98.

35. Claassens DMF, Vos GJA, Bergmeijer TO, Hermanides RS, van 't Hof AWJ, van der Harst P, et al. A Genotype-Guided Strategy for Oral P2Y 12 Inhibitors in Primary PCI. N Engl J Med [Internet]. 2019 Oct 24;381(17):1621–31.

36. Pereira NL, Farkouh ME, So D, Lennon R, Geller N, Mathew V, et al. Effect of Genotype-Guided Oral P2Y12 Inhibitor Selection vs Conventional Clopidogrel Therapy on Ischemic Outcomes After Percutaneous Coronary Intervention. JAMA [Internet]. 2020 Aug 25;324(8):761.

For peer review only

Figure 1: UK Biobank participants eligible for analysis: cohort flowchart

Cohort flowchart shows how the eligible participants to study were identified. Summary statistics for the 7,483 participants are available in Table 1. *Participants never prescribed clopidogrel were used for sensitivity analysis of CYP2C19 LoF variants and vascular outcomes.

For peer review only

For peer review only - http://bmjopen.bmj.com/site/about/guidelines.xhtml

Figure 2: Kaplan-Meier plots of strokes and MIs in patients prescribed clopidogrel, stratified by CYP2C19 Loss of Function genotypes

Kaplan-Meier failure plots for A) incident ischaemic stroke and B) incident MI showing cumulative hazard (%) with increasing time taking clopidogrel in patients prescribed clopidogrel for at least 2 months, stratified by CYP2C19 genotype (carriers of any *2-*8 Loss of Function variant, “intermediate/poor metabolizers of clopidogrel,” vs. *1/*1 normal metabolizers).

For peer review only

For peer review only - http://bmjopen.bmj.com/site/about/guidelines.xhtml

Table 1: Summary of UK Biobank participants with GP-prescribed clopidogrel

	CYP2C19 genotype	
	**Normal metabolizer (*1/*1)**	**Intermediate/ poor (any *2-*8)**
Number of participants (% of total n=7,483)	5,338 (71.3)	2,145 (28.7)
Females, n (% of genotype group)	1,847 (34.6)	712 (33.2)
Age at first clopidogrel prescription		
Minimum : maximum	36.5 to 78.8	37.6 to 78.1
Mean (SD)	64.1 (7.3)	64.3 (7.2)
Diagnoses* prior to clopidogrel prescription, n (%)		
Ischaemic stroke	509 (9.5)	240 (11.2)
Transient ischaemic attack	1,142 (21.4)	422 (19.7)
Myocardial infarction / angina	2,980 (55.8)	1,267 (59.1)
Atrial fibrillation	408 (7.6)	180 (8.4)
Hypertension	3,072 (57.6)	1,277 (59.5)
Peripheral vascular disease	442 (8.3)	177 (8.3)
Any of the above six diagnoses	4,863 (91.1)	1,982 (92.4)
Number of clopidogrel prescriptions		
Minimum : maximum	2 to 467.0	2 to 494.0
Mean (SD)	28.3 (36.7)	27.9 (36.9)
Years between first and last clopidogrel prescription		
Minimum : maximum	0.2 to 17.9	0.2 to 17.3
Mean (SD)	2.6 (3.0)	2.5 (2.9)
Also prescribed aspirin whilst taking clopidogrel, n (%)	2,632 (49.3)	1,119 (52.2)
Ischaemic stroke whilst taking clopidogrel, n (%)	69 (1.3)	41 (1.9)
Myocardial infarction whilst taking clopidogrel, n (%)	1,268 (24.1)	554 (26.4)

*Analysis of European-ancestry participants with >1 clopidogrel prescription in the available GP prescribing data. Participants excluded if clopidogrel prescribing frequency was less than once every 2 months. Events occurring after the last known date of clopidogrel prescription are also excluded. * Ischaemic stroke diagnoses are from hospital in-patient records only; all other disease also include diagnoses recorded in primary care.*

For peer review only - http://bmjopen.bmj.com/site/about/guidelines.xhtml

1 **Table 2: *CYP2C19* genotype associations with incident ischaemic stroke and MI events in patients prescribed clopidogrel**

**Outcome, model**	***CYP2C19 genotype***	**N**	**N cases**	**Person-years**	**HR**	**95% CIs**		***p***
**Ischaemic stroke**								
All participants	Normal metabolizer (*1/*1)	5,333	69	13,248				
	Intermediate/poor (any *2-*8)	2,144	41	5,110	1.53	1.04	2.26	0.031
	Total	*7,477*	*110*	*18,358*				
Events in first year only	Normal metabolizer (*1/*1)	5,333	25	4,419				
	Intermediate/poor (any *2-*8)	2,144	14	1,789	1.33	0.69	2.57	0.390
	Total	*7,477*	*39*	*6,208*				
Events after first year only	Normal metabolizer (*1/*1)	3,071	44	8,829				
	Intermediate/poor (any *2-*8)	1,245	27	3,322	1.63	1.01	2.64	0.047
	Total	*4,316*	*71*	*12,151*				
**Myocardial Infarction**								
All participants	Normal metabolizer (*1/*1)	5,259	1,269	10,093				
	Intermediate/poor (any *2-*8)	2,100	554	3,709	1.14	1.04	1.26	0.008
	Total	*7,359*	*1,823*	*13,803*				
Events in first year only	Normal metabolizer (*1/*1)	5,255	906	3,831				
	Intermediate/poor (any *2-*8)	2,099	415	1,511	1.16	1.03	1.31	0.011
	Total	*7,354*	*1,321*	*5,342*				
Events after first year only	Normal metabolizer (*1/*1)	2,529	362	6,258				
	Intermediate/poor (any *2-*8)	982	139	2,198	1.09	0.90	1.33	0.370
	Total	*3,511*	*501*	*8,455*				

Analysis of European-ancestry participants with >1 clopidogrel prescription in the available GP prescribing data. Participants excluded if clopidogrel prescribing frequency was less than once every 2 months. Events <1 week after first clopidogrel prescription are excluded. Events occurring after the last known date of clopidogrel prescription are also excluded. HR=Hazard Ratios from Cox's proportional hazards regression models adjusted for age at first clopidogrel prescription, sex, and genetic principal components of ancestry 1-10. CI = Confidence Intervals.

For peer review only

1

For peer review only

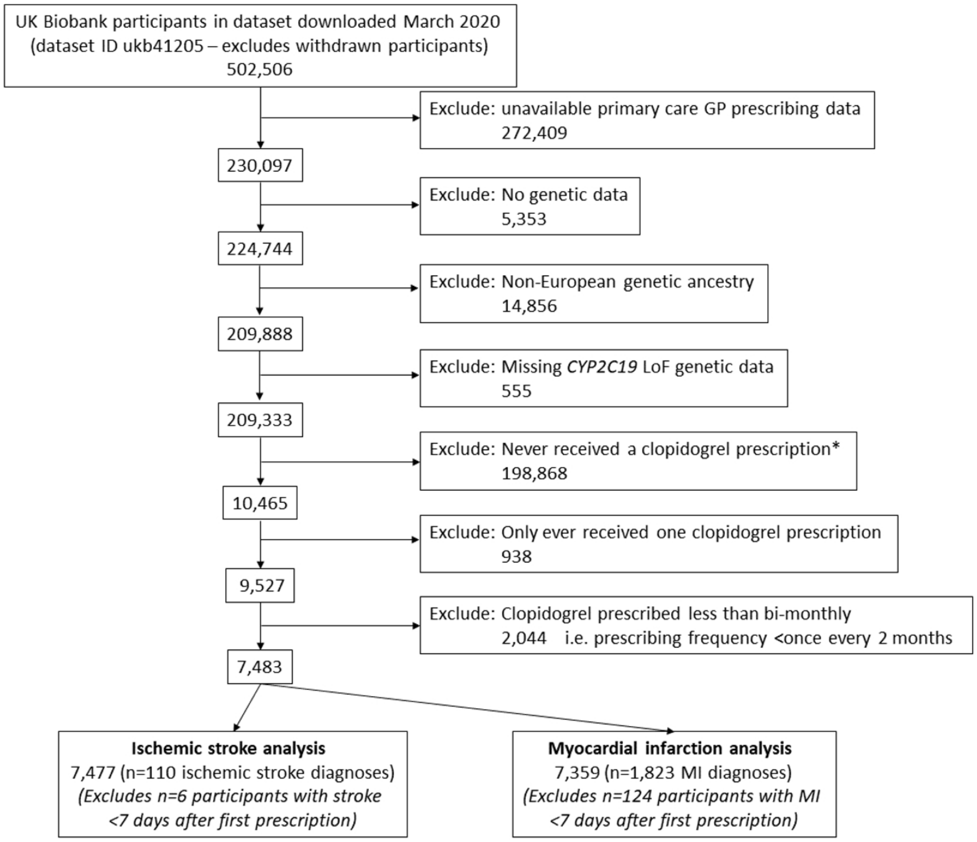

Figure 1: UK Biobank participants eligible for analysis: cohort flowchart
Cohort flowchart shows how the eligible participants to study were identified. Summary statistics for the 7,483 participants are available in Table 1. *Participants never prescribed clopidogrel were used for sensitivity analysis of CYP2C19 LoF variants and vascular outcomes.

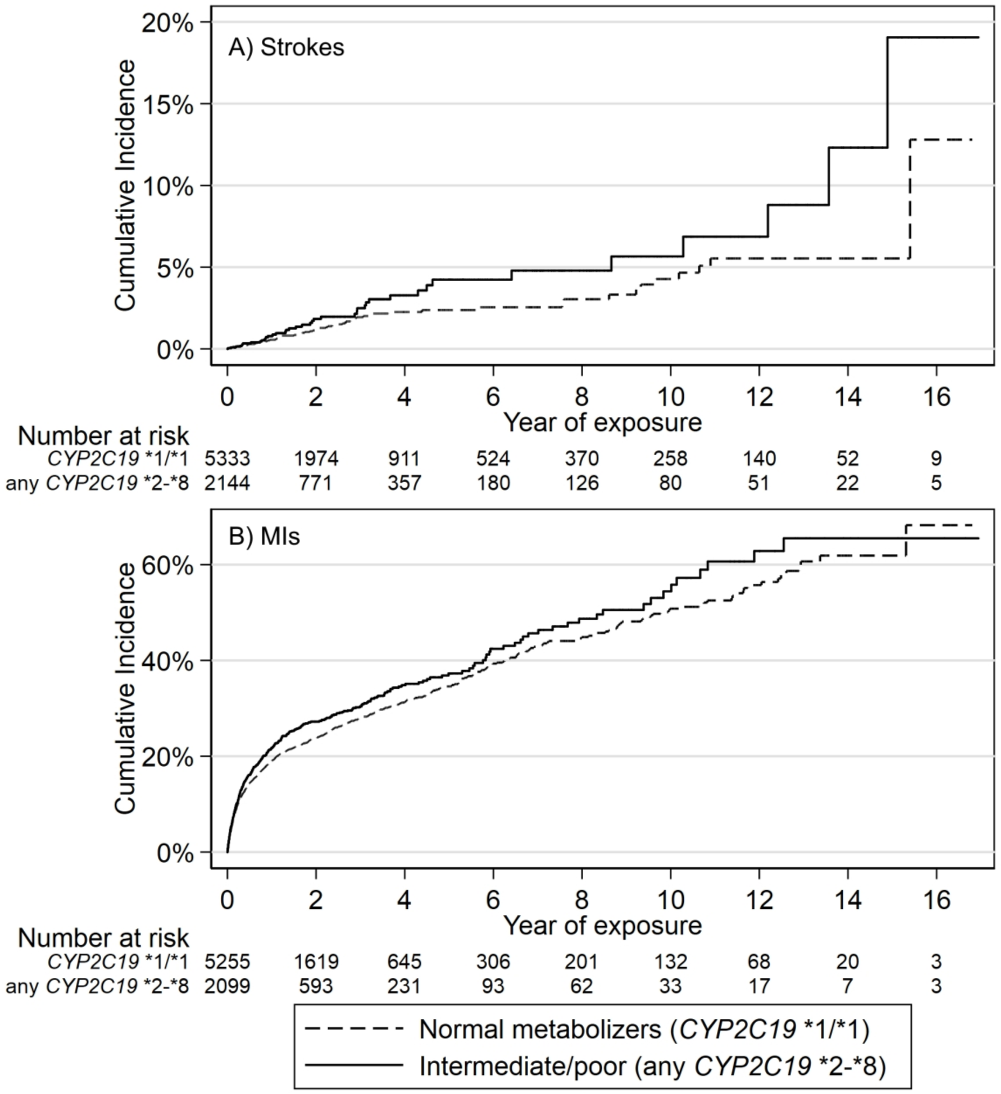

Figure 2: Kaplan-Meier plots of strokes and MIs in patients prescribed clopidogrel, stratified by CYP2C19 Loss of Function genotypes

Kaplan-Meier failure plots for A) incident ischaemic stroke and B) incident MI showing cumulative hazard (%) with increasing time taking clopidogrel in patients prescribed clopidogrel for at least 2 months, stratified by CYP2C19 genotype (carriers of any *2-*8 Loss of Function variant, "intermediate/poor metabolizers of clopidogrel," vs. *1/*1 normal metabolizers).

SUPPLEMENTARY MATERIAL

Analysis of *CYP2C19* genetic variants with ischaemic events in UK patients prescribed clopidogrel in primary care: a retrospective cohort study

Pilling *et al*.

Contents

Supplementary Methods 1
Prescription data 1
Supplementary Table 1: prescription codes in UK Biobank 2
Supplementary Table 2: *CYP2C19* genotypes in UK Biobank 3
Supplementary Table 3: ICD-10 codes for Major Bleeding outcome 4
Supplementary Table 4: *CYP2C19* associations with incident outcomes stratified by *17 genotype 5

Supplementary Methods

Prescription data

To identify prescriptions of clopidogrel and aspirin we used the UK Biobank “Coding system lookups and mappings” file (https://biobank.ctsu.ox.ac.uk/crystal/refer.cgi?id=592). For clopidogrel we searched for “clopidogrel|plavix|grepid” to include other relevant drug names. For aspirin we searched for “aspirin|nu-seals|caprin|disprin” also including relevant alternate names. We then used the associated read 2 codes and drug names in the `gp_scripts` UK Biobank prescription data for the participants. We did not use BNF codes as the resolution was not great enough to uniquely identify individual drugs.

For peer review only - http://bmjopen.bmj.com/site/about/guidelines.xhtml

Supplementary Table 1: prescription codes in UK Biobank

medication	term_description	read_2	bnf_code
clopidogrel	clopidogrel	bu5..	02.09.00.00
	clopidogrel 300mg tablets	bu54.	02.09.00.00
	clopidogrel 75mg tablets	bu51.	02.09.00.00
	grepid 75mg tablets	bu55.	02.09.00.00
	plavix 300mg tablets	bu53.	02.09.00.00
	plavix 75mg tablets	bu52.	02.09.00.00
aspirin	*aspirin 100mg m/r tablets	bu29.	02.09.00.00
	*aspirin 300mg m/r tablets	bu2b.	02.09.00.00
	*aspirin 324mg e/c tablets	di1h.	04.07.01.00
	*aspirin 500mg m/r tablets	di19.	
	*aspirin 600mg e/c tablets	di1g.	04.07.01.00
	*aspirin 600mg tablets	di1i.	00.00.00.00
	*aspirin 75mg tablets	bu25.	02.09.00.00
	*caprin 300mg e/c tablets	di1k.	04.07.01.00
	*caprin 324mg e/c tablets	di1a.	04.07.01.00
	*caprin 75mg e/c tablets	bu2F.	02.09.00.00
	*disprin cv 100mg m/r tablets	bu28.	02.09.00.00
	*disprin cv 300mg m/r tablets	bu2a.	02.09.00.00
	aspirin 100mg effervescent tablets	bu21.	02.09.00.00
	aspirin 150mg suppositories	di1o.	04.07.01.00
	aspirin 162.5mg m/r capsules	bu2I.	02.09.00.00
	aspirin 300mg dispersible tablets	j112.	10.01.01.00
	aspirin 300mg e/c tablets	di1f.	04.07.01.00
	aspirin 300mg effervescent tablets	bu27.	02.09.00.00
	aspirin 300mg soluble tablets	di1m.	04.07.01.00
	aspirin 300mg suppositories	di1n.	04.07.01.00
	aspirin 300mg tablets	j111.	10.01.01.00
	aspirin 75mg dispersible tablets	di13.	
	aspirin 75mg dispersible tablets	bu23.	02.09.00.00
	aspirin 75mg e/c tablets	bu2B.	02.09.00.00
	aspirin 75mg soluble tablets	bu2c.	02.09.00.00
	aspirin [antiplatelet]	bu2..	02.09.00.00
	aspirin [central nervous system use]	di1..	04.07.01.00
	aspirin [cns] 300mg dispersible tablets	di12.	04.07.01.00
	aspirin [cns] 300mg tablets	di11.	04.07.01.00
	aspirin [musculoskeletal use]	j11..	10.01.01.00
	aspirin and the salicylates	j1...	10.01.01.00
	aspirin+metoclopramide 900mg/10mg/sachet powder	dl1b.	04.07.04.01
	aspirin+papaveretum 500mg/7.71mg dispersible tablets	diaG.	04.07.01.00
	aspirin/paracetamol/codeine tablets	dia1.	04.07.01.00
	dipyridamole+aspirin	bu4..	02.09.00.00
	dipyridamole+aspirin 200mg/25mg m/r capsules	bu41.	02.09.00.00
	disprin 300mg dispersible tablets	di1r.	04.07.01.00
	isosorbide mononitrate+aspirin	blm..	02.06.01.00
	isosorbide mononitrate+aspirin 60mg/150mg m/r tablets	blmy.	02.06.01.00
	isosorbide mononitrate+aspirin 60mg/75mg m/r tablets	blmz.	02.06.01.00
	nu-seals aspirin 300mg e/c tablets	di1c.	04.07.01.00
	nu-seals aspirin 600mg e/c tablets	di1d.	04.07.01.00
	nu-seals aspirin 75mg e/c tablets	bu2A.	02.09.00.00
	nu-seals cardio 75 e/c tablets	bu2G.	02.09.00.00

For peer review only

For peer review only - http://bmjopen.bmj.com/site/about/guidelines.xhtml

Supplementary Table 2: *CYP2C19* genotypes in UK Biobank

							UK Biobank						GnomAD	
**CYP2C19**	**Function**	**RSID**	**CHR**	**BP**	**A1**	**A2**	**HWE_p**	**INFO**	**MAF1**	**MAF1 %**	**note**		**MAF2**	**MAF2 %**
*2	LoF	rs4244285	10	96541616	G	A	0.9067	0.9998	0.1492	14.924	Imputed		0.1468	14.6800
*3	LoF	rs4986893	10	96540410	G	A	1.0000	1.0000	0.0001	0.006	Directly genotyped		0.0003	0.0264
*4	LoF	rs28399504	10	96522463	A	G	0.1615	0.7339	0.0022	0.216	Imputed		0.0025	0.2528
*5	LoF	rs56337013	10	96612495	C	T					Not in UKB imputed		0.0000	0.0008
*6	LoF	rs72552267	10	96535210	G	A					Not in UKB imputed		0.0003	0.0333
*7	LoF	rs72558186	10	96541756	T	C					Not in UKB imputed		0.0000	0.0000
*8	LoF	rs41291556	10	96535173	T	C	0.0415	1.0000	0.0030	0.304	Directly genotyped		0.0027	0.2710
*17	GoF	rs12248560	10	96521657	C	T	0.7279	0.9984	0.2154	21.539	Imputed		0.2314	23.1400

Function = LoF (Loss of Function, poor metaboliser), GoF (Gain of Function, rapid metaboliser)
BP = base position (hg19, build 37)
A1 = common allele
A2 = minor allele
HWE_p = Hardy-Weinberg deviation p-value
INFO = imputation quality score
MAF1 = minor allele frequency in UK Biobank Europeans
MAF2 = GnomAD European (non-Finnish) population minor allele frequency. GnomAD data can be viewed using URLs https://gnomad.broadinstitute.org/variant/rs4244285 and substituting the RSID for the appropriate variant.

For peer review only

Supplementary Table 3: ICD-10 codes for Major Bleeding outcome

From DOI 10.1111/jep.13400

Code	Text Descriptor	Event
D62	Acute post-haemorrhagic anaemia	Other major bleeding
H35.6	Retinal haemorrhage	Other major bleeding
H43.1	Vitreous haemorrhage	Other major bleeding
I60.*	Subarachnoid haemorrhage	Intracranial bleeding
I61.*	Intracerebral haemorrhage	Intracranial bleeding
I62.*	Other non-traumatic intracranial haemorrhage	Intracranial bleeding
I85.0	Oesophageal varices with bleeding	GI bleeding
J94.2	Haemothorax	Other major bleeding
K25.0	Gastric ulcer, acute with haemorrhage	GI bleeding
K25.2	Gastric ulcer, acute with both haemorrhage and perforation	GI bleeding
K25.4	Gastric ulcer, chronic or unspecified with haemorrhage	GI bleeding
K25.6	Chronic or unspecified with both haemorrhage and perforation	GI bleeding
K26.0	Duodenal ulcer, acute with haemorrhage	GI bleeding
K26.2	Duodenal ulcer, acute with both haemorrhage and perforation	GI bleeding
K26.4	Duodenal ulcer, chronic or unspecified with haemorrhage	GI bleeding
K26.6	Chronic or unspecified with both haemorrhage and perforation	GI bleeding
K27.0	Peptic ulcer, acute with haemorrhage	GI bleeding
K27.2	Peptic ulcer, acute with both haemorrhage and perforation	GI bleeding
K27.4	Peptic ulcer, chronic or unspecified with haemorrhage	GI bleeding
K27.6	Chronic or unspecified with both haemorrhage and perforation	GI bleeding
K28.0	Gastrojejunal ulcer, acute with haemorrhage	GI bleeding
K28.2	Acute with both haemorrhage and perforation	GI bleeding
K28.6	Chronic or unspecified with both haemorrhage and perforation	GI bleeding
K29.0	Acute haemorrhagic gastritis	GI bleeding
K66.1	Haemoperitoneum	GI bleeding
K92.0	Haematemesis	GI bleeding
K92.1	Melaena	GI bleeding
N02.*	Recurrent and persistent haematuria	Other major bleeding
N92.4	Excessive bleeding in the premenopausal period	Other major bleeding
R04.*	Haemorrhage from respiratory passages	Other major bleeding
R58	Haemorrhage, not elsewhere classified	Other major bleeding

Note: we excluded N95.0, K62.5 and R31.* from our criteria as these were common (>10,000 participants ever diagnosed in whole dataset) and therefore deemed not specific to Major Bleeding for the purpose of this analysis.

For peer review only

Supplementary Table 4: *CYP2C19* associations with incident outcomes stratified by *17 genotype

Outcome / CYP2C19 genotype	N	N cases	Person-years	HR	95% CIs		*p*
Ischemic stroke							
Normal (*1/*1)	2,948	37	7,208				
Intermediate/poor (any *2-*8)	1,668	33	3,919	1.61	1.01	2.58	0.047
Poor or rapid heterozygote (*2–*8/*17)	476	8	1,191	1.25	0.58	2.69	0.570
Rapid (any *17)	2,385	32	6,039	0.99	0.62	1.59	0.970
Total	*7,477*	*110*	*18,358*				
Myocardial infarction							
Normal (*1/*1)	2,905	707	5,403				
Intermediate/poor (any *2-*8)	1,634	442	2,805	1.16	1.03	1.30	0.017
Poor or rapid heterozygote (*2–*8/*17)	466	112	904	0.98	0.81	1.20	0.880
Rapid (any *17)	2,354	562	4,691	0.95	0.85	1.06	0.330
Total	*7,359*	*1823*	*13,803*				

Analysis of European-ancestry participants with >1 clopidogrel prescription in the available GP prescribing data. Participants excluded if clopidogrel prescribing frequency was less than once every 2 months. Events <1 week after first clopidogrel prescription are excluded. Events occurring after the last known date of clopidogrel prescription are also excluded. HR=Hazard Ratios from Cox's proportional hazards regression models adjusted for age at first clopidogrel prescription, sex, and genetic principal components of ancestry 1-10. CI = Confidence Intervals.

For peer review only - http://bmjopen.bmj.com/site/about/guidelines.xhtml

STROBE 2007 (v4) Statement—Checklist of items that should be included in reports of *cohort studies*

Section/Topic	Item #	Recommendation	Reported on page #
**Title and abstract**	1	(*a*) Indicate the study's design with a commonly used term in the title or the abstract	1,2
		(*b*) Provide in the abstract an informative and balanced summary of what was done and what was found	2
**Introduction**			
Background/rationale	2	Explain the scientific background and rationale for the investigation being reported	4
Objectives	3	State specific objectives, including any prespecified hypotheses	4,5
**Methods**			
Study design	4	Present key elements of study design early in the paper	4-7
Setting	5	Describe the setting, locations, and relevant dates, including periods of recruitment, exposure, follow-up, and data collection	6
Participants	6	(*a*) Give the eligibility criteria, and the sources and methods of selection of participants. Describe methods of follow-up	6-8, Figure 1 (flowchart)
		(*b*) For matched studies, give matching criteria and number of exposed and unexposed	Na
Variables	7	Clearly define all outcomes, exposures, predictors, potential confounders, and effect modifiers. Give diagnostic criteria, if applicable	6-9
Data sources/ measurement	8*	For each variable of interest, give sources of data and details of methods of assessment (measurement). Describe comparability of assessment methods if there is more than one group	6-8
Bias	9	Describe any efforts to address potential sources of bias	8-9
Study size	10	Explain how the study size was arrived at	6-8
Quantitative variables	11	Explain how quantitative variables were handled in the analyses. If applicable, describe which groupings were chosen and why	8-9 (time to event, rather than quantitative)
Statistical methods	12	(*a*) Describe all statistical methods, including those used to control for confounding	8-9
		(*b*) Describe any methods used to examine subgroups and interactions	8-9
		(*c*) Explain how missing data were addressed	6-8, Figure 1 (flowchart)
		(*d*) If applicable, explain how loss to follow-up was addressed	Na

For peer review only

		(*e*) Describe any sensitivity analyses	8-9
**Results**			
Participants	13*	(a) Report numbers of individuals at each stage of study—eg numbers potentially eligible, examined for eligibility, confirmed eligible, included in the study, completing follow-up, and analysed	10, Figure 1 (cohort flowchart)
		(b) Give reasons for non-participation at each stage	10, Figure 1 (cohort flowchart)
		(c) Consider use of a flow diagram	Figure 1
Descriptive data	14*	(a) Give characteristics of study participants (eg demographic, clinical, social) and information on exposures and potential confounders	10. Table 1
		(b) Indicate number of participants with missing data for each variable of interest	10, Figure 1 (cohort flowchart)
		(c) Summarise follow-up time (eg, average and total amount)	10. Table 1
Outcome data	15*	Report numbers of outcome events or summary measures over time	10-12. Table 1 & 2
Main results	16	(*a*) Give unadjusted estimates and, if applicable, confounder-adjusted estimates and their precision (eg, 95% confidence interval). Make clear which confounders were adjusted for and why they were included	10-13. Absolute effects are unadjusted. Regression estimates adjusted for age and sex.
		(*b*) Report category boundaries when continuous variables were categorized	Na
		(*c*) If relevant, consider translating estimates of relative risk into absolute risk for a meaningful time period	10, 12
Other analyses	17	Report other analyses done—eg analyses of subgroups and interactions, and sensitivity analyses	10-12
**Discussion**			
Key results	18	Summarise key results with reference to study objectives	14
**Limitations**			
Interpretation	20	Give a cautious overall interpretation of results considering objectives, limitations, multiplicity of analyses, results from similar studies, and other relevant evidence	14-15
Generalisability	21	Discuss the generalisability (external validity) of the study results	15
**Other information**			
Funding	22	Give the source of funding and the role of the funders for the present study and, if applicable, for the original study on	16

For peer review only

		which the present article is based	

*Give information separately for cases and controls in case-control studies and, if applicable, for exposed and unexposed groups in cohort and cross-sectional studies.

Note: An Explanation and Elaboration article discusses each checklist item and gives methodological background and published examples of transparent reporting. The STROBE checklist is best used in conjunction with this article (freely available on the Web sites of PLoS Medicine at http://www.plosmedicine.org/, Annals of Internal Medicine at http://www.annals.org/, and Epidemiology at http://www.epidem.com/). Information on the STROBE Initiative is available at www.strobe-statement.org.

For peer review only
